# Selective Dopamine Transporter Inhibition with CE-123 Promotes Fear Extinction and Alters the Basolateral Amygdala Proteome Following Fear Extinction in Adolescent Male Rats

**DOI:** 10.3390/ijms27188352

**Published:** 2026-09-19

**Authors:** Pawel Grochecki, Malgorzata Hopcias, Justyna Lubinska, Tymoteusz Slowik, Gert Lubec, Joanna Listos, Piotr Suder, Jolanta H. Kotlinska

**Affiliations:** 1 Department of Pharmacology and Pharmacodynamics, Medical University of Lublin, Chodzki 4a st., 20-093 Lublin, Poland; pawel.grochecki@umlub.edu.pl (P.G.); d.justynalubinska@umlub.edu.pl (J.L.); joanna.listos@umlub.edu.pl (J.L.); 2 Department of Analytical Chemistry and Biochemistry, Faculty of Materials Sciences and Ceramics, AGH University of Krakow, Mickiewicza 30 Avenue, 30-059 Krakow, Poland; hopcias@agh.edu.pl (M.H.); piotr.suder@agh.edu.pl (P.S.); 3 Experimental Medicine Center, Medical University, Jaczewskiego 8, 20-090 Lublin, Poland; tymoteusz.slowik@umlub.edu.pl; 4 Department of Neuroproteomics, Paracelsus Medical University, 5020 Salzburg, Austria; gert.lubec@lubeclab.com

**Keywords:** fear extinction, dopamine transporter, CE-123, basolateral amygdala, hippocampus, proteomics

## Abstract

Persistent fear responses and impaired fear extinction are characteristic features of stress-related disorders. Dopaminergic signalling within amygdala-centred circuits contributes to fear extinction, but the associated molecular mechanisms remain unclear. Here, we investigated whether selective dopamine transporter (DAT) inhibition with CE-123 (10 mg/kg, intraperitoneally) facilitates fear extinction and induces region-specific molecular adaptations. Adolescent male rats were subjected to a modified stress-enhanced fear learning (SEFL)-like paradigm followed by extinction training. Rats exposed to high-intensity fear conditioning (STRESSED groups) displayed persistent freezing and increased anxiety-like behaviour compared with non-shocked controls (NON-STRESSED groups). Repeated CE-123 administration during extinction significantly reduced freezing in stressed rats without affecting non-stressed animals. Quantitative proteomic analyses of the basolateral amygdala (BLA) and hippocampus (HPC) revealed marked region-specific effects. Stress-related fear enhancement induced extensive proteomic remodelling in the BLA, involving proteins associated with glutamatergic signalling, synaptic plasticity, and axonal organization, whereas the hippocampal proteome was largely unaffected. CE-123 also produced BLA-specific molecular effects, reducing the abundance of proteins associated with mitochondrial ATP synthesis in non-stressed animals and with ribosomal, mitochondrial, and RNA-processing pathways in stressed rats. These findings identify the BLA as a key substrate of fear-related molecular adaptations and demonstrate that selective DAT inhibition modulates amygdala proteomic architecture during fear extinction. Overall, the results support further investigation of DAT modulation as a potential strategy for facilitating fear extinction in stress-related disorders.

## 1. Introduction

Exposure to highly aversive events can induce persistent alterations in emotional learning, including exaggerated threat responses, impaired fear extinction, and increased anxiety-like behavior. These processes are mediated by interconnected neural circuits involving the amygdala, hippocampus (HPC), and prefrontal cortex (PFC), which regulate the acquisition, expression, extinction, and updating of emotional memories. Impaired fear extinction is a characteristic feature of several stress-related psychiatric disorders, including post-traumatic stress disorder (PTSD), anxiety disorders, and specific phobias [1,2]. Currently available pharmacological treatments for PTSD, including the selective serotonin reuptake inhibitors sertraline and paroxetine, show limited efficacy, particularly in chronic cases, and relapse remains common [3,4,5]. These limitations highlight the need for novel approaches targeting the neural mechanisms that support extinction learning and adaptive memory updating.

Adolescence is characterized by the continued maturation of cortical, limbic, and striatal circuits involved in emotional regulation, cognitive flexibility, and motivated behavior [6,7]. During this period, dopaminergic neurotransmission undergoes substantial remodeling within mesocortical and mesolimbic pathways, alongside the refinement of prefrontal–amygdala–hippocampal circuitry [6,7]. The concurrent development of these neural circuits and stress-responsive systems may increase vulnerability to stressful experiences and their persistent effects on emotional learning [7,8]. Given the heightened neural plasticity characteristic of adolescence, this developmental period may also provide an important framework for investigating how pharmacological modulation of dopaminergic signaling influences fear extinction.

Among the neurotransmitter systems involved in fear regulation, dopamine is a critical modulator of emotional learning, behavioral flexibility, and memory updating [9,10]. Fear extinction represents new inhibitory learning initiated when an expected aversive unconditioned stimulus (US) is omitted, generating a positive prediction-error signal. In mice, ventral tegmental area (VTA) dopamine neurons are transiently activated by unexpected US omission during early extinction, and the magnitude of this response predicts the subsequent reduction in freezing. Accordingly, temporally precise inhibition of these neurons at the time of US omission impairs extinction, whereas their activation accelerates extinction learning [11]. This effect is circuit- and cell population-specific: VTA dopamine projections to reward-responsive Ppp1r1b-positive neurons in the posterior basolateral amygdala (BLA) promote extinction, whereas activation of projections to fear-responsive Rspo2-positive neurons in the anterior BLA can impair extinction [12]. Dopamine neurons also encode threat-prediction certainty, and their phasic activation can prevent or reverse fear generalization [13]. Furthermore, recall of a reward-associated cue has been shown to suppress fear generalization and enhance extinction through dopamine-dependent modulation of central amygdala circuits [14]. Together, these findings indicate that dopamine contributes to updating fear memories when threat contingencies change. Clinical and preclinical studies have also demonstrated alterations in dopamine transporter (DAT) expression and dopamine receptor signaling following traumatic stress, suggesting that dysregulated dopaminergic neurotransmission may contribute to persistent fear and impaired extinction [15,16,17,18,19]. Selective DAT inhibition may therefore provide a means of facilitating dopamine-dependent extinction processes.

CE-123 (5-((benzhydrylsulfinyl)methyl)thiazole) is a modafinil-related compound that selectively inhibits DAT while showing minimal affinity for norepinephrine and serotonin transporters [20]. Unlike classical psychostimulants, CE-123 moderately increases extracellular dopamine without producing hyperlocomotion, stereotypic behavior, or reinforcing effects. It also improves attention, executive function, and cognitive flexibility in preclinical studies [21,22,23,24]. These properties make CE-123 a useful pharmacological tool for examining whether selective DAT inhibition can facilitate extinction-related memory updating. However, the molecular adaptations associated with its potential effects on fear extinction remain poorly understood, particularly at the level of specific brain regions involved in fear regulation.

Experimental fear-conditioning paradigms provide robust models for studying the neural and molecular mechanisms of fear acquisition and extinction. Although they do not reproduce the full clinical phenotype of PTSD, they model discrete processes relevant to stress-related psychopathology, including persistent conditioned fear, impaired extinction, fear generalization, and enhanced responses to subsequent aversive experiences [25]. In the present study, we used a modified stress-enhanced fear learning (SEFL)-like paradigm in which an initial high-intensity footshock was followed by a low-intensity reminder procedure, providing a framework for examining persistent fear and its extinction without repeated severe aversive exposure.

In the present study, we investigated whether repeated administration of CE-123 during extinction training facilitates the extinction of fear acquired in early adolescence (postnatal day 30; PND30), as assessed in young adult male rats (PND50–51), and produces region-specific proteomic adaptations. Proteomic analyses focused on the BLA and HPC because these regions have complementary roles in fear processing: the BLA is critical for forming and updating associations between conditioned and aversive stimuli, whereas the HPC contributes to contextual representation and discrimination. We hypothesized that CE-123 would facilitate fear extinction in stressed animals and that its behavioral effects would be accompanied by molecular adaptations, particularly within the BLA.

## 2. Results

### 2.1. Effect of Conditioning with an Aversive Electric Stimulus on Freezing Time in Response to a Moderate-Intensity Stimulus and to the Conditioned Stimulus

To determine whether aversive conditioning induced sensitization to a weaker aversive stimulus and persistent fear responses, freezing behavior was assessed during the fear reminder session in a novel context (context B) on PND42. On PND43, fear memory recall was evaluated under shock-free conditions in both the original conditioning context (context A) and the reminder context (context B) to assess the persistence and generalization of conditioned fear.

**Figure 1:**
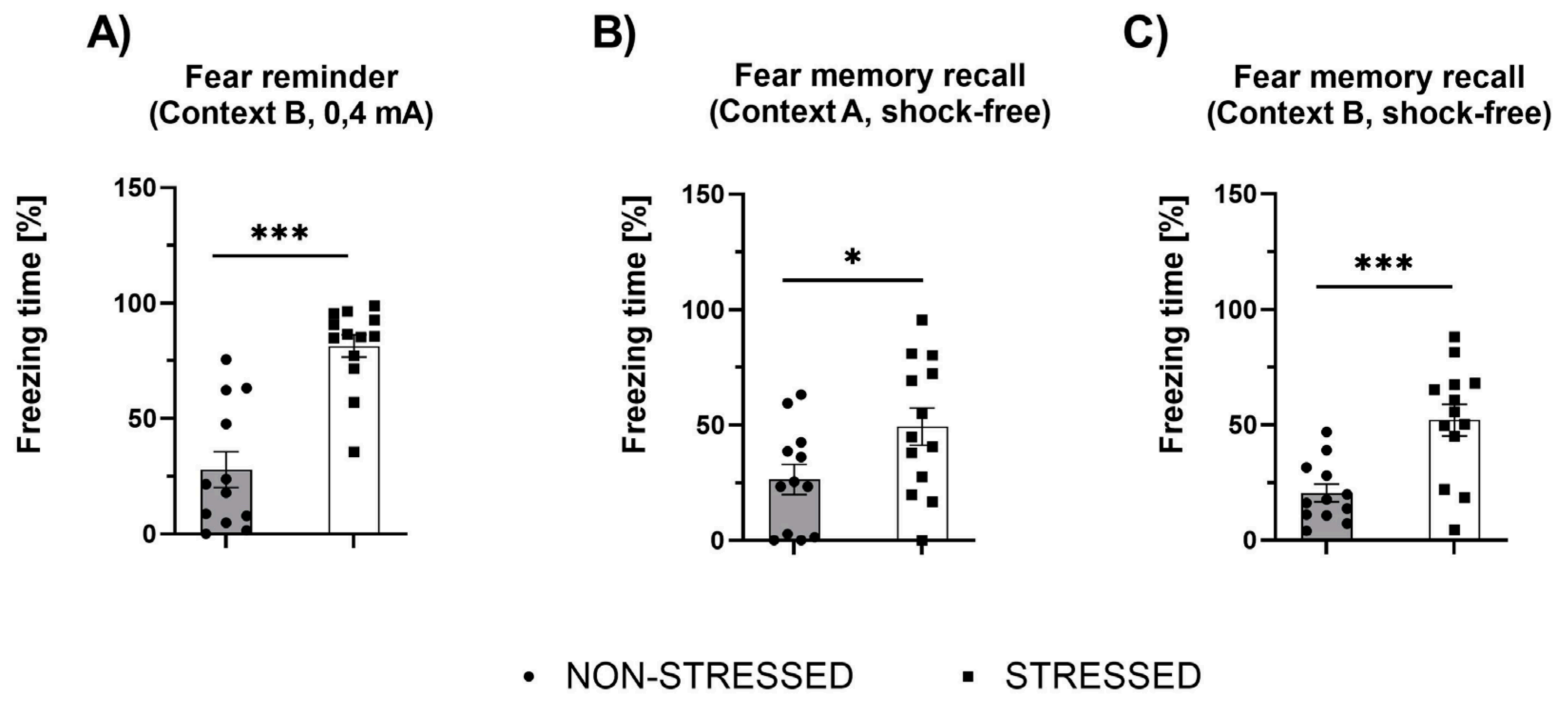
Freezing behavior during the fear reminder session and fear memory recall. (**A**) Fear reminder in Context B following exposure to mild foot shocks (0.4 mA). (**B**) Fear memory recall in Context A (shock-free). (**C**) Fear memory recall in Context B (shock-free). Data are presented as mean ± SEM. Individual animals are shown as circles (NON-STRESSED) and squares (STRESSED). * *p* < 0.05, *** *p* < 0.001. Individual animals are shown as circles (NON-STRESSED, *n* = 12) and squares (STRESSED, *n* = 13). * *p* < 0.05, *** *p* < 0.001.

Exposure to a strong aversive stimulus (1.5 mA) on PND30 in context A sensitized animals to a weaker aversive stimulus (0.4 mA), resulting in a significant increase in freezing measured on PND42 in context B. Freezing was significantly higher in the STRESSED group than in the NON-STRESSED group (81.47% vs. 27.96%; unpaired Student’s *t*-test, t(23) = 5.888, *p* < 0.0001; Figure 1A).

Thirteen days after conditioning, STRESSED animals continued to exhibit significantly higher freezing than NON-STRESSED animals in both the conditioning context (context A: 49.35% vs. 26.40%; t(23) = 2.197, *p* = 0.0384; Figure 1B) and the novel context (context B: 52.05% vs. 20.55%; t(23) = 3.938, *p* = 0.0007; Figure 1C). These findings indicate persistent, conditioned fear in the original conditioning context and generalized fear responses in a novel context.

### 2.2. Effect of Conditioning with an Aversive Electric Stimulus on Anxiety-like Behavior in the EPM Test

To determine whether aversive conditioning produced persistent anxiety-like behavior beyond conditioned freezing, animals were assessed in the EPM on PND44, before the initiation of CE-123 treatment. The percentage of time spent in the open arms and the percentage of open-arm entries were evaluated as measures of anxiety-like behavior, whereas the total distance traveled was used to assess overall exploratory activity during the test.

**Figure 2:**
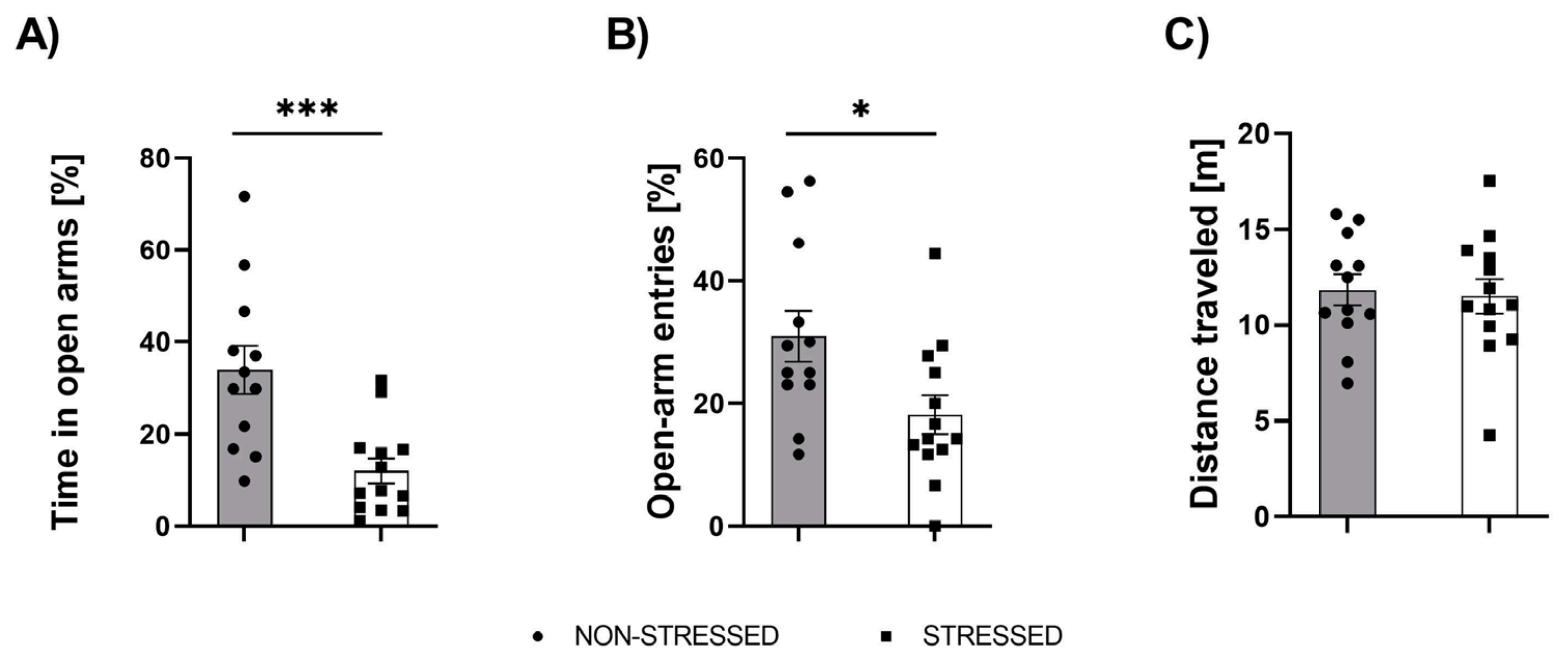
Elevated plus maze (EPM) behavior following aversive fear conditioning. (**A**) Percentage of time spent in the open arms relative to the total test duration, (**B**) percentage of open-arm entries relative to the total number of arm entries, and (**C**) total distance traveled. Data are presented as mean ± SEM (NON-STRESSED, *n* = 12; STRESSED, *n* = 13). Individual animals are shown as circles (NON-STRESSED) and squares (STRESSED). * *p* < 0.05, *** *p* < 0.001.

Exposure to a strong aversive stimulus (1.5 mA) on PND30 significantly increased anxiety-like behavior measured in the elevated plus maze (EPM) on PND44. Compared with the NON-STRESSED group, STRESSED animals showed a significantly lower percentage of time spent in the open arms (12.08% vs. 33.89%; unpaired Student’s *t*-test, t(23) = 3.819, *p* = 0.0009; Figure 2A) and a lower percentage of open-arm entries (18.16% vs. 30.99%; t(23) = 2.484, *p* = 0.0207; Figure 2B), indicating reduced exploration of the open arms.

In contrast, the total distance traveled during the EPM test did not differ significantly between the STRESSED and NON-STRESSED groups (11.50 vs. 11.83 m; t(23) = 0.2685, *p* = 0.7907; Figure 2C).

### 2.3. Effect of Repeated CE-123 Administration During Aversive Memory Extinction on Freezing Time in Response to a Low-Intensity Stimulus and the Conditioned Stimulus

To determine whether repeated CE-123 administration during extinction training affected the subsequent expression of conditioned fear, freezing was assessed in STRESSED and NON-STRESSED animals receiving either CE-123 or vehicle. Post-extinction behavior was evaluated under two conditions: during a fear reinstatement session involving the conditioned auditory stimulus and low-intensity foot shocks on PND50, and during a shock-free final test involving the conditioned auditory stimulus on PND51.

**Figure 3:**
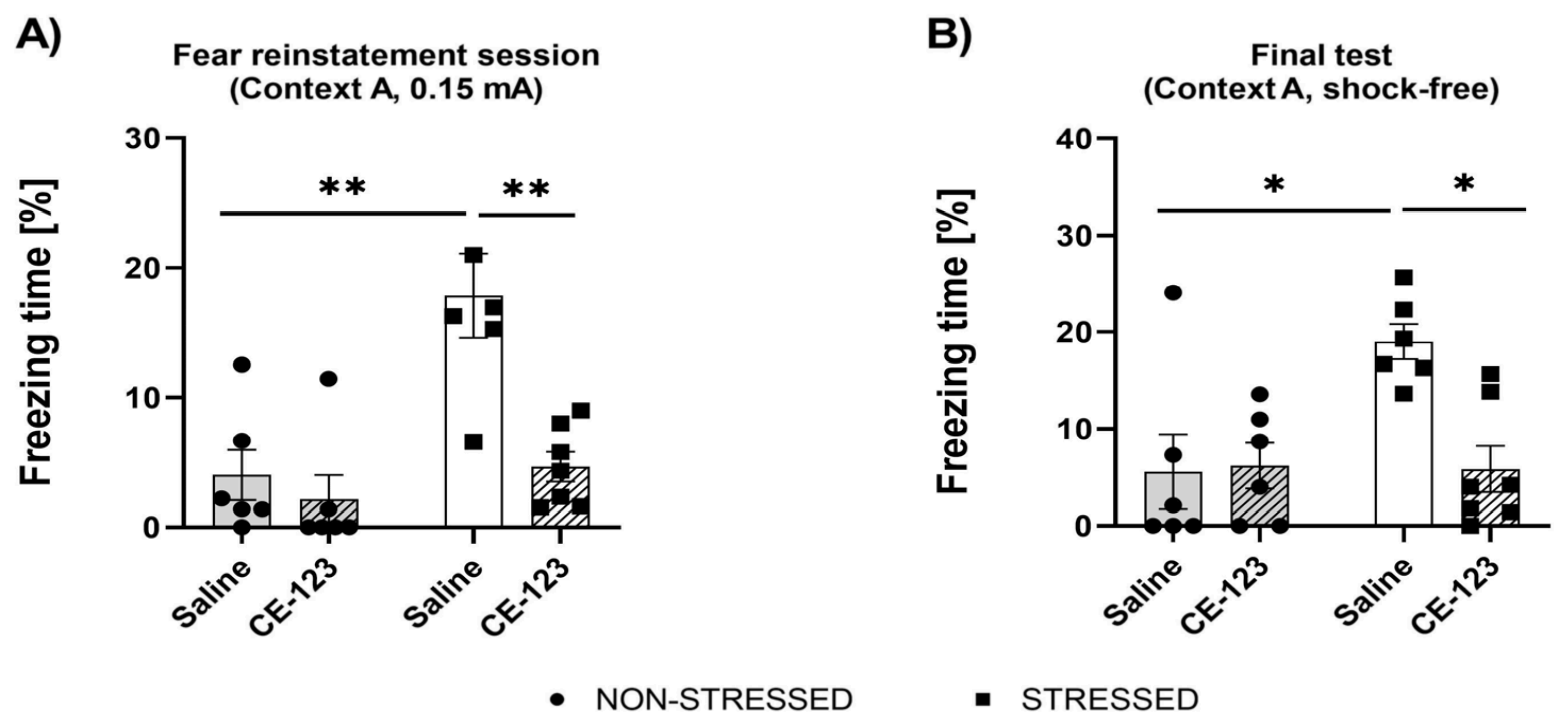
Effect of repeated CE-123 administration (10 mg/kg, i.p.) during fear extinction on freezing behavior during the post-extinction fear reinstatement session (**A**) and the final shock-free test (**B**). The fear reinstatement session was conducted in context A and included low-intensity footshocks (0.15 mA), whereas the final test was conducted in context A without footshock. Data are presented as mean ± SEM (NON-STRESSED + saline, *n* = 6; NON-STRESSED + CE-123, *n* = 6; STRESSED + saline, *n* = 6; STRESSED + CE-123, *n* = 7). Individual animals are shown as circles (NON-STRESSED) and squares (STRESSED). * *p* < 0.05, ** *p* < 0.01.

On PND50, during the post-extinction fear reinstatement session, two-way ANOVA revealed significant main effects of stress [F(1, 21) = 14.66, *p* < 0.01] and repeated CE-123 administration during the extinction phase [F(1, 21) = 12.50, *p* < 0.01], as well as a significant stress × treatment interaction [F(1, 21) = 7.021, *p* < 0.05]. Bonferroni’s post hoc test showed significantly higher freezing in saline-treated STRESSED animals than in saline-treated NON-STRESSED animals (*p* < 0.01), whereas repeated CE-123 administration significantly reduced freezing in STRESSED animals (*p* < 0.01; Figure 3A). CE-123-treated STRESSED animals did not differ from CE-123-treated NON-STRESSED animals (*p* > 0.05).

Similarly, during the shock-free final test on PND51, two-way ANOVA revealed significant main effects of stress [F(1, 21) = 5.863, *p* < 0.05] and repeated CE-123 administration [F(1, 21) = 5.391, *p* < 0.05], together with a significant stress × treatment interaction [F(1, 21) = 6.526, *p* < 0.05]. Bonferroni’s post hoc test showed significantly higher freezing in saline-treated STRESSED animals than in saline-treated NON-STRESSED animals (*p* < 0.05), whereas repeated CE-123 administration during extinction significantly reduced freezing in STRESSED animals (*p* < 0.05; Figure 3B). CE-123-treated STRESSED animals did not differ from CE-123-treated NON-STRESSED animals (*p* > 0.05), indicating that CE-123 prevented the stress-associated increase in freezing during the final test.

### 2.4. Proteomic Investigations

To identify region-specific molecular alterations associated with persistent fear and the behavioral effects of CE-123, proteomic analyses were performed in the HPC and BLA, two brain regions critically involved in fear-memory processing and extinction. Tissue samples were collected immediately after the final behavioral assessment on PND51, and the experimental comparisons were designed to distinguish stress-related alterations from the effects of CE-123 in stressed and non-stressed animals.

Proteomic analyses were performed for three pairwise comparisons: (1) STRESSED vs. NON-STRESSED, (2) STRESSED + CE-123 vs. STRESSED, and (3) NON-STRESSED + CE-123 vs. NON-STRESSED. All analyses were performed independently for the HPC and BLA.

#### 2.4.1. Limited Proteomic Remodeling in the HPC

**Figure 4:**
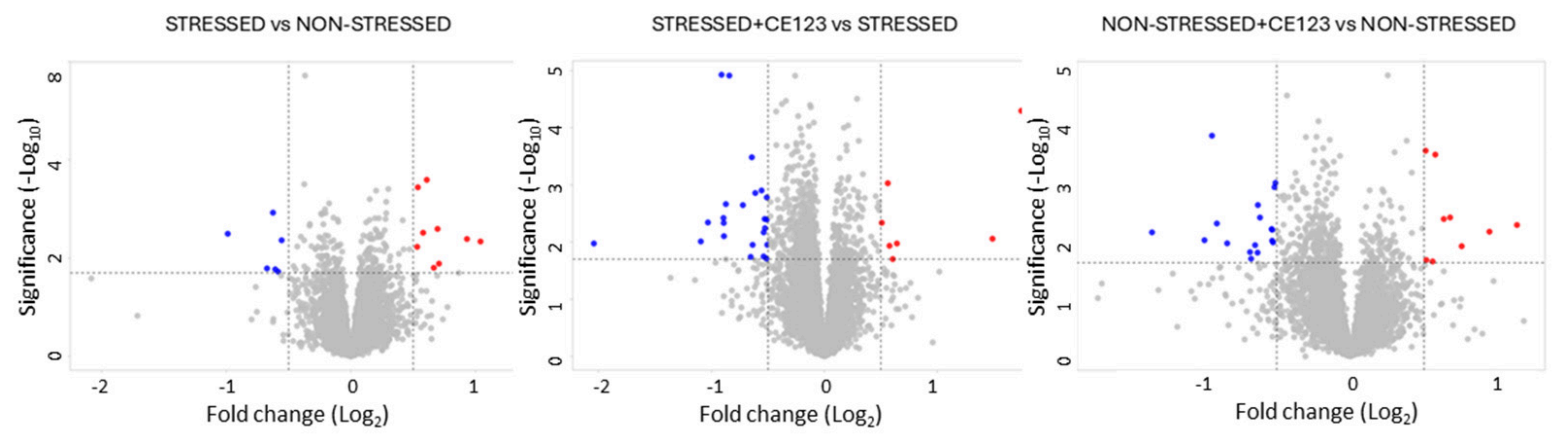
Volcano plots illustrating differential protein abundance in the hippocampus (HPC) across all pairwise comparisons. Red and blue dots indicate proteins with increased and decreased abundance, respectively. For visualization purposes, volcano plots were generated using a threshold of *p* = 0.02 (−log_10_(*p*) = 1.7) and an absolute log_2_(fold change) > 0.5. Statistical significance was determined using FDR-adjusted analyses as described in the Methods. Detailed results, including protein names, gene names, log_2_(fold change), raw *p*-values, and FDR-adjusted *p*-values, are provided in Supplementary Table S1.

The hippocampal proteome showed only limited changes across the experimental groups. On average, 4,500–4,800 proteins were identified per sample. Comparison of the NON-STRESSED and STRESSED groups revealed only nine upregulated and six downregulated proteins (Figure 4, left panel; Table S1), according to the predefined statistical criteria. STRING analysis did not identify significant protein–protein interaction enrichment. Although several differentially expressed proteins were associated with neuronal plasticity (e.g., Dock9, Ptbp3, and Cplx2) or cellular metabolism (e.g., Foxred1, Clybl, and Prkcd), they did not form coherent functional interaction networks.

Similarly, comparison of the STRESSED + CE-123 and STRESSED groups revealed only a small number of differentially expressed proteins (Figure 4, middle panel; Table S1). STRING analysis identified only small interaction clusters related to protein folding, structural stabilization, and mitochondrial metabolism. Likewise, no pronounced or functionally convergent proteomic alterations were observed in the NON-STRESSED + CE-123 and NON-STRESSED comparison (Figure 4, right panel).

These findings indicate that, at this time point, the hippocampal proteome undergoes only limited remodeling following stress exposure or CE-123 administration. Volcano plots for all hippocampal comparisons are shown in Figure 4, and the corresponding lists of differentially expressed proteins are provided in Table S1.

#### 2.4.2. Extensive Proteomic Remodeling in the BLA

**Figure 5:**
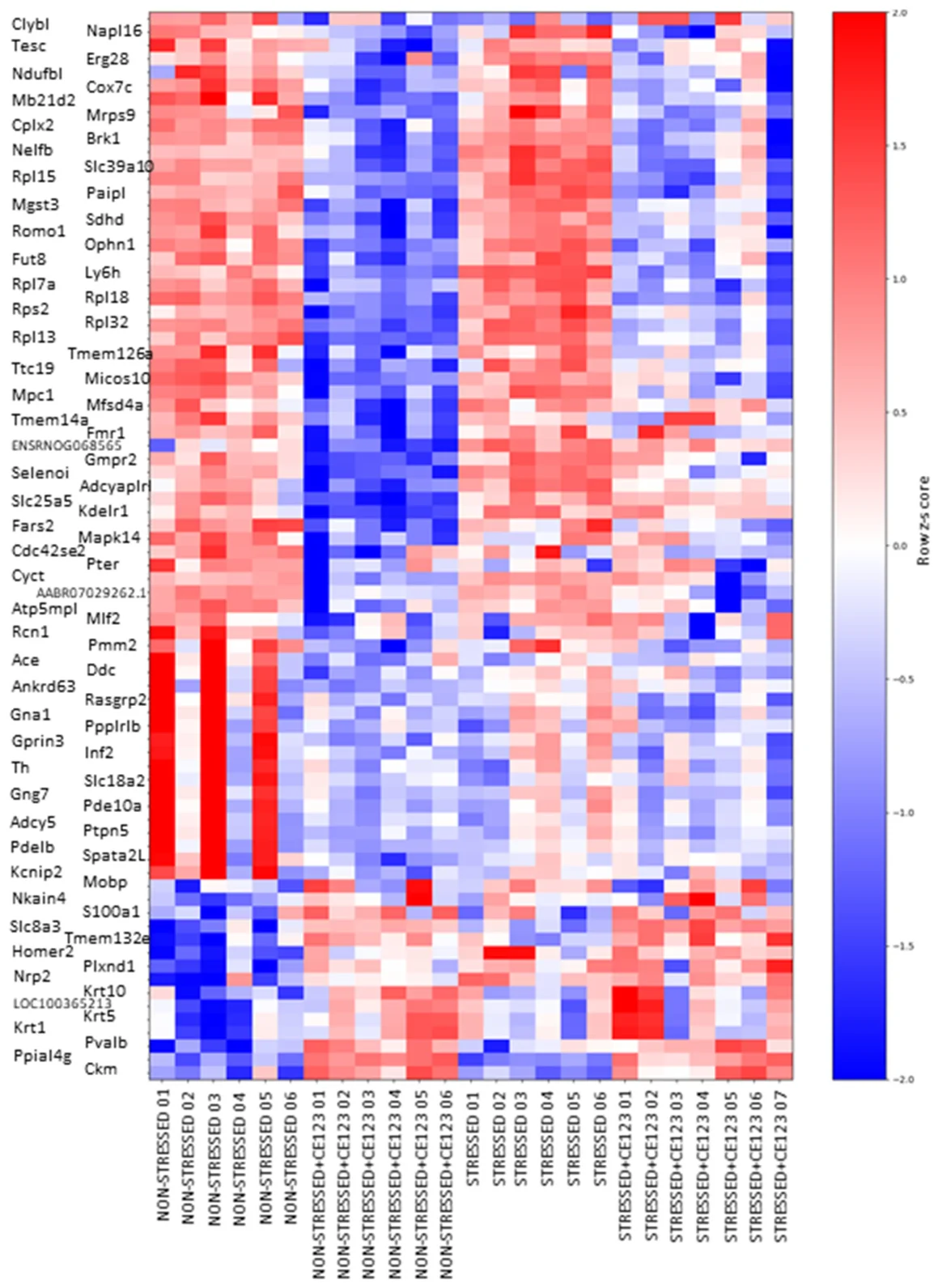
Heatmap illustrating CE-123-induced proteomic changes in the basolateral amygdala (BLA) across all experimental groups. The top 80 differentially abundant proteins were selected based on the highest absolute log2(fold change) between the NON-STRESSED and NON-STRESSED + CE-123 groups. Protein abundance values are presented as row-wise z-scores across all samples. Hierarchical clustering separated proteins into two major clusters with opposite abundance patterns associated with CE-123 treatment. The NON-STRESSED + CE-123 group exhibited a distinct protein abundance profile compared with the NON-STRESSED group, whereas the STRESSED and STRESSED + CE-123 groups showed partially overlapping but distinguishable expression patterns.

Figure 5 presents a heatmap of the top 80 differentially abundant proteins in the BLA, demonstrating distinct CE-123-associated protein abundance patterns and hierarchical clustering that differentiate the experimental groups.

**Figure 6:**
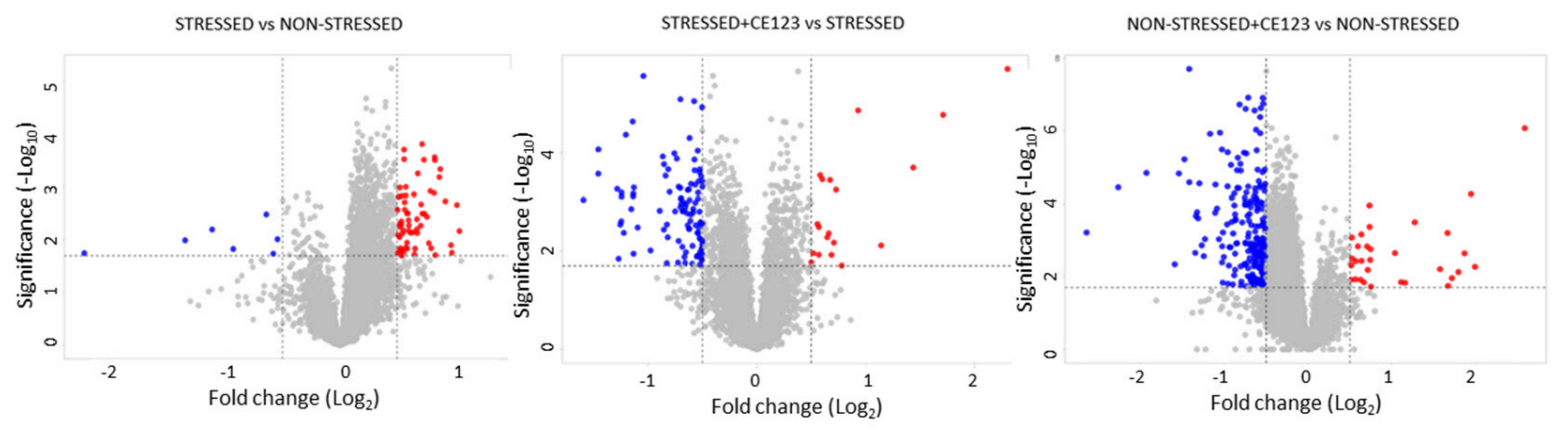
Volcano plots illustrating differential protein abundance in the basolateral amygdala (BLA) across all pairwise comparisons. Red and blue dots indicate proteins with increased and decreased abundance, respectively. For visualization purposes, volcano plots were generated using a threshold of *p* = 0.02 (−log_10_(*p*) = 1.7) and an absolute log_2_(fold change) > 0.5. Statistical significance was determined using FDR-adjusted analyses as described in the Methods. Detailed results, including protein names, gene names, log_2_(fold change), raw *p*-values, and FDR-adjusted *p*-values, are provided in Supplementary Table S2.

In contrast to the HPC, the BLA displayed extensive proteomic alterations. Comparison of the STRESSED and NON-STRESSED groups identified 54 differentially abundant proteins, including 48 upregulated and 6 downregulated proteins (Figure 6, left panel, Table S2). Gene Ontology (GO) biological process enrichment analysis revealed significant overrepresentation of proteins involved in axonal development and nervous system development. Cellular component analysis demonstrated enrichment of proteins associated with glutamatergic synapses, including postsynaptic signaling components, with sixteen proteins localized predominantly to postsynaptic regions (Figure S1). Within the postsynaptic and glutamatergic categories enriched in the STRESSED versus NON-STRESSED comparison, representative proteins showing increased abundance included GRIA4, HOMER2, and CAMK2D. GRIA4 is an AMPA receptor subunit involved in excitatory glutamatergic transmission, HOMER2 acts as a postsynaptic scaffolding protein, and CAMK2D participates in calcium-dependent signalling and synaptic plasticity [26,27,28]. KIF5A, DNER, NRP2, and PLXND1 were also upregulated and are associated with axonal transport, neuronal differentiation, axon guidance, and neuronal circuit organization [29,30]. The corresponding log_2_(fold change) and FDR-adjusted *p*-values are provided in Supplementary Table S2.

Comparison of the STRESSED + CE-123 and STRESSED groups identified 97 differentially abundant proteins, including 81 downregulated and 16 upregulated proteins following CE-123 administration (Figure 6, middle panel, Table S2). GO molecular function enrichment analysis revealed enrichment of RNA-binding proteins among the downregulated proteins. In addition, downregulated proteins were associated with mitochondrial function, GTP hydrolysis, and RNA metabolism (Figure S2).

To evaluate the effects of CE-123 in the absence of stress, the NON-STRESSED + CE-123 and NON-STRESSED groups were compared. CE-123 treatment resulted in differential abundance of 200 proteins, predominantly downregulated (174 downregulated and 26 upregulated proteins; Figure 6, right panel, Table S2). Enrichment analysis revealed underrepresentation of proteins involved in mitochondrial ATP synthesis and electron transport chain processes, including multiple components of the respirasome (e.g., Cox6a1, Cox7a2, Cox6c, Sdhc, HIGd1a, ND3, and Dmac2) (Figure S3).

The most significantly altered proteins are summarized in Table 1, whereas the complete lists of differentially abundant proteins are provided in Supplementary Tables S1 and S2. Overall, proteomic alterations were substantially more pronounced in the BLA than in the HPC.

To further assess the overall structure of the proteomic datasets, principal component analysis (PCA) was performed for both brain regions (Figure S4). BLA samples showed a clear separation between the experimental groups, whereas HPC samples exhibited markedly weaker clustering, consistent with the limited proteomic alterations observed in this structure. In addition, overlap analysis of stress-regulated proteins in the BLA and HPC (Figure S5) identified only five commonly regulated proteins, all showing the same direction of change, indicating a limited overlap between the proteomic responses of the two brain regions.

## 3. Discussion

The present study showed that exposure to an intense aversive stimulus produced persistent fear- and anxiety-like behavior in adolescent rats. Animals subjected to foot-shock conditioning displayed increased freezing in response to both a subsequent weak aversive stimulus and the conditioned auditory cue, together with reduced open-arm exploration in the EPM. A high-intensity unconditioned stimulus paired with a conditioned cue can induce long-lasting fear in rodents, whereas a low-intensity reminder shock delivered in a novel context is commonly used in SEFL-like procedures to reactivate and strengthen the original fear memory rather than produce de novo conditioning [1,25,31]. Accordingly, the combination of high-intensity conditioning and a mild reminder stimulus produced persistent and generalized fear responses relevant to impaired fear regulation in stress-related disorders. Although the applied paradigm does not reproduce the full clinical phenotype of PTSD, it provides an appropriate experimental framework for investigating persistent fear and impaired extinction.

A major finding of the present study was that repeated administration of CE-123 during extinction training reduced freezing in stressed animals during both post-extinction assessments. On PND50, freezing was measured under compound test conditions involving re-exposure to context A, presentation of the conditioned auditory stimulus, and delivery of a low-intensity foot shock. On PND51, freezing was assessed in context A during a shock-free test involving the conditioned auditory stimulus. Consequently, the PND50 assessment does not allow the respective contributions of the contextual, auditory, and mild aversive components to be distinguished. Nevertheless, the reduction in freezing observed in CE-123-treated stressed animals under both testing conditions, including the shock-free assessment on PND51, is consistent with enhanced extinction-related suppression of conditioned fear.

Accumulating evidence indicates that dopaminergic mechanisms play an important role in prediction-error signalling and the updating of threat-related memories. In particular, the unexpected omission of an anticipated aversive stimulus during extinction evokes phasic activity of VTA dopamine neurons, thereby promoting the formation of a new safety memory [11]. Further studies have indicated that this effect is mediated, at least partly, through functionally distinct dopaminergic projections to the BLA and that dopaminergic signalling within amygdala-centred circuits can influence both fear generalization and extinction [12,13,14]. By enhancing dopaminergic transmission through DAT inhibition, CE-123 may therefore have strengthened prediction-error processing, facilitated the incorporation of new safety information, and promoted cognitive flexibility during extinction [18,20]. However, because dopaminergic signalling and the activity of specific VTA–BLA circuits were not directly assessed, this mechanistic interpretation remains hypothetical.

When interpreting the behavioral effects of CE-123, the possibility of nonspecific changes in locomotor activity or behavioral activation should also be considered. The absence of a difference in the total distance travelled during the EPM between the STRESSED and NON-STRESSED groups indicates that their differences in open-arm measures were unlikely to result from altered general exploratory activity. However, the EPM was performed before CE-123 administration commenced and therefore cannot be used to evaluate the effects of the compound on locomotion. Previous studies indicate that CE-123 administered at 10 mg/kg does not induce psychostimulant-like hyperactivity or substantially alter spontaneous locomotor activity [21,22,24]. Nevertheless, because no dedicated locomotor assessment was performed during the CE-123 treatment period, subtle effects on arousal, motivation, or behavioral engagement during extinction training cannot be excluded.

At the molecular level, the proteomic analyses revealed a marked regional difference between the HPC and BLA. Relatively few differentially abundant proteins were detected in the HPC, whereas substantially more extensive proteomic alterations occurred in the BLA. The limited hippocampal response should not be interpreted as evidence that the HPC was not involved in the contextual processing or extinction of fear. Instead, extensive changes in total hippocampal protein abundance were not detectable at the single, relatively late sampling point used in this study, approximately three weeks after conditioning and immediately after the final behavioral assessment. Hippocampal involvement may have depended on transient molecular responses occurring during acquisition, consolidation, retrieval, or extinction that were no longer detectable at tissue collection. Moreover, bulk-tissue proteomics may obscure subregion-, cell-type-, and synaptic compartment-specific responses within the anatomically and functionally heterogeneous HPC. Functionally relevant changes in protein phosphorylation, localization, or activity may also occur without corresponding alterations in total protein abundance. Thus, under the present experimental and analytical conditions, proteomic remodeling was substantially more pronounced in the BLA than in the HPC, without excluding an important functional contribution of the HPC to contextual fear processing.

Stress-related proteomic remodeling in the BLA was associated with biological processes involving axonal and nervous system development, glutamatergic synaptic organization, and postsynaptic compartments. Among the upregulated proteins, GRIA4, HOMER2, and CAMK2D represent functionally related components of excitatory neurotransmission and activity-dependent plasticity. GRIA4 is a subunit of AMPA receptors, which mediate rapid excitatory glutamatergic transmission and undergo activity-dependent trafficking and compositional changes during synaptic plasticity [26,27,32]. Stress-induced enhancement of fear-memory consolidation has also been linked to increased synaptic mobilization of AMPA receptors within amygdala circuits [28]. HOMER2 functions as a postsynaptic scaffolding protein that organizes signalling complexes involving glutamate receptors and intracellular effector pathways [33,34], whereas CAMK2D participates in calcium-dependent signalling relevant to synaptic plasticity. Their coordinated upregulation is therefore consistent with altered excitatory postsynaptic organization in the BLA following persistent fear conditioning.

A second group of upregulated proteins, comprising KIF5A, DNER, NRP2, and PLXND1, is associated with the structural organization and development of neuronal circuits. KIF5A is a neuronally enriched motor protein involved in anterograde axonal transport, including the transport of organelles and macromolecular cargoes required for neuronal maintenance [29,35]. DNER, NRP2, and PLXND1 are associated with neuronal development, semaphorin-related signalling, axon guidance, and the organization of neuronal connectivity [30,36]. Together, these findings suggest that persistent fear was accompanied by changes in both excitatory synaptic signalling and the structural organization of BLA networks. These alterations are consistent with the established role of amygdala-centred circuits in fear-memory formation and regulation [37,38]. However, because protein abundance was measured only after completion of conditioning, extinction training, and post-extinction testing, the detected changes cannot be attributed exclusively to fear acquisition or persistence and may also incorporate molecular responses to subsequent extinction-related experiences.

CE-123 produced a distinct pattern of proteomic alterations in the BLA rather than simply reversing the profile observed in stressed animals. In the STRESSED + CE-123 versus STRESSED comparison, most differentially abundant proteins were downregulated and were enriched in pathways associated with RNA binding and metabolism, mitochondrial function, and GTP hydrolysis. This pattern suggests that CE-123 induced a broader reorganization of processes related to protein synthesis, cellular metabolism, and neuronal function. Proteins directly involved in dopamine synthesis, metabolism, or receptor signalling were not prominently represented among the differentially abundant proteins. The observed changes may therefore reflect downstream molecular adaptations to DAT inhibition rather than direct alterations of the core dopaminergic machinery.

Several proteins upregulated following CE-123 administration may be relevant to neuronal excitability and activity-dependent plasticity. Functional annotation indicated that PVALB, JPH4, and SLC8A3 are associated with inhibitory neuronal function, intracellular calcium handling, and ion homeostasis, whereas CPLX2, GRID2, MAP2K2, and OPHN1 are linked to synaptic vesicle release, glutamate receptor-related signalling, intracellular signal transduction, and neuronal structural organization (Table S2). Although their precise contributions cannot be determined from bulk proteomic data, their coordinated alteration suggests modulation of processes controlling neuronal excitability and synaptic organization within BLA networks. These changes may reflect increased molecular flexibility of BLA circuits during or following extinction learning. However, the present data do not demonstrate that these proteins directly mediated the behavioral effects of CE-123, and their functional significance will require targeted validation.

CE-123 also altered the abundance of proteins associated with mitochondrial ATP synthesis and oxidative phosphorylation, including components of the respiratory chain such as COX6A1, COX7A2, and SDHC. Neuronal activity and synaptic plasticity place substantial demands on mitochondrial energy metabolism; consequently, these changes may represent metabolic adaptations accompanying altered neuronal activity. Importantly, mitochondrial and electron-transport-chain-related changes were also observed after CE-123 administration in non-stressed animals. Modulation of cellular energy metabolism may therefore represent a broader pharmacological effect of CE-123 rather than a stress-specific mechanism of facilitated extinction. Whether these metabolic changes contribute to extinction-related plasticity or constitute a parallel response to repeated DAT inhibition remains to be established.

Previous evidence that subchronic S-CE-123 administration altered the synaptosomal proteome of the PFC in aged rats [23], considered together with the present BLA findings, suggests that the compound can affect molecular pathways across multiple brain regions involved in cognitive and emotional regulation. However, the functional categories associated with CE-123 differed among the PFC, BLA, and HPC, suggesting a region-specific molecular response. In the present study, the behavioral and proteomic findings converge in identifying the BLA as a potentially important substrate of the response associated with CE-123-facilitated fear extinction. Nevertheless, the proteomic changes should be interpreted as molecular associations accompanying the behavioral effect rather than as evidence of a causal mechanism.

Several limitations should be acknowledged. First, the study was conducted exclusively in adolescent male rats. Sex differences influence vulnerability to stress-related disorders, fear extinction, and the underlying neurobiological mechanisms [39]. The present findings may therefore not generalize to females, and future studies should include both sexes and evaluate potential sex-dependent behavioral and proteomic responses to CE-123.

Second, locomotor activity was not assessed during CE-123 treatment, and proteomic analyses were limited to the BLA and HPC at a single post-extinction time point. Thus, subtle treatment-related changes in behavioral activation cannot be excluded, while the involvement of other fear-related regions, particularly the medial prefrontal cortex (mPFC), and the temporal dynamics of the molecular responses remain to be determined.

Finally, the proteomic findings were not validated using independent methods such as Western blotting, immunohistochemistry, or targeted mass spectrometry. The identified protein changes should therefore be considered exploratory and hypothesis-generating. Functional studies involving targeted manipulation of selected candidates will be required to determine whether they contribute causally to the effects of CE-123 on fear extinction.

## 4. Materials and Methods

### 4.1. Animal Maintenance and Treatment

The study was conducted on adolescent male Wistar rats (PND30 at the start of the experiment; total *n* = 25) bred and raised at the certified institutional breeding facility of the Medical University of Lublin (OMD, Lublin, Poland). Approximately one week before the beginning of the experiment, the animals were transferred to the experimental housing area within the University and allowed to acclimatize to the housing and environmental conditions before any experimental procedures were initiated. Animals were initially assigned to two experimental conditions: NON-STRESSED (*n* = 12) and STRESSED (*n* = 13). During the fear extinction procedure, when CE-123 administration was initiated, animals within each condition were further subdivided into treatment groups, resulting in the following group sizes: NON-STRESSED (*n* = 6), NON-STRESSED + CE-123 (*n* = 6), STRESSED (*n* = 6), and STRESSED + CE-123 (*n* = 7). Animals were housed in standard polypropylene cages (55 × 33 × 20 cm) in the vivarium of the Medical University of Lublin under controlled laboratory conditions, with a temperature of 22 ± 1 °C, relative humidity of 55 ± 10%, and a 12 h light/dark cycle (lights on at 8:00 a.m.). Standard laboratory chow (Sniff Spezialdiäten GmbH, Soest, Germany) and tap water were available ad libitum.

All experimental procedures were conducted in accordance with the National Institutes of Health Guide for the Care and Use of Laboratory Animals and the applicable European legislation for the protection of animals used for scientific purposes. The experimental protocol was approved by the Local Ethics Committee (approval no. 11/2024). Every effort was made to minimize animal suffering and to reduce the number of animals used.

### 4.2. Chemicals

The S-enantiomer of CE-123 (5-((benzhydrylsulfinyl)methyl)thiazole), a modafinil derivative, was used in the present study. The compound was generously provided by the Lubec Laboratory (University of Vienna, Austria). S-CE-123 belongs to the class of thiazole derivatives and was synthesized according to published protocols, yielding high enantiomeric and chemical purity (99.7%, determined by HPLC analysis), making it suitable for in vivo experiments [23]. On each day of administration, CE-123 was freshly prepared by suspending it in a vehicle containing 1% DMSO and 3.3% Tween 80 (both from Sigma-Aldrich, St. Louis, MO, USA) in 0.9% NaCl (Natrium chloratum 0.9% Kabi; Fresenius Kabi Polska Sp. z o.o., Warsaw, Poland).

CE-123 or its vehicle was administered intraperitoneally (i.p.) once daily at a dose of 10 mg/kg during the fear extinction phase (PND 46–49). The dose of 10 mg/kg and the intraperitoneal route of administration were selected based on previous studies demonstrating that CE-123 at this dose effectively improved learning, memory, and cognitive flexibility in rats, including adolescent animals and experimental models of neurodevelopmental impairment [20,21,22,23,24]. Pharmacokinetic studies have also confirmed that CE-123 administered intraperitoneally at 10 mg/kg reaches the brain at measurable concentrations, supporting central exposure at the selected dose [40]. CE-123 remains an experimental compound without an established clinical dose; therefore, direct correspondence between the dose used in the present study and human exposure cannot currently be determined.

Animals were randomly assigned to four experimental groups: Group 1 (NON-STRESSED; *n* = 6) received vehicle (i.p.) during the extinction phase; Group 2 (NON-STRESSED + CE-123; *n* = 6) received CE-123 (10 mg/kg, i.p.); Group 3 (STRESSED; *n* = 6) received vehicle (i.p.); and Group 4 (STRESSED + CE-123; *n* = 7) received CE-123 (10 mg/kg, i.p.) (Table 2). Behavioral testing was conducted between PND 30 and PND 51.

### 4.3. Behavioral Apparatus and Procedures

#### 4.3.1. Fear Conditioning Apparatus

A fear conditioning apparatus was used to induce the fear conditioning model [41]. During conditioning, rats were placed in a soundproof chamber (Ugo Basile, Italy; 55 × 60 × 57 cm) equipped with a light source, a speaker, and a metal-bar floor for the delivery of foot shocks. The procedure was conducted as described previously [41].

#### 4.3.2. Aversive Stimulus Conditioning (Context A) (PND30)

During the conditioning session, the neutral (conditioned) stimulus (CS) consisted of a 30-s auditory tone (80 dB) delivered through a loudspeaker inside the chamber (context A). In the final 2 s of the tone, a 1.5 mA foot shock was administered as the aversive unconditioned stimulus (US). After a 30-s interval, the tone–shock pairing was presented a second time. Once the second pairing ended, the rat remained in the chamber for an additional 30 s to allow memory consolidation before being returned to its home cage.

Before introducing the next animal, the chamber was disinfected with medical-grade ethanol to remove any residual olfactory cues. The odour of the cleaning agent, together with the metal-grid floor (10 mm spacing) and plain chamber walls, constituted the contextual elements defining context A. The entire conditioning protocol lasted 150 s (2.5 min) and followed this sequence: 30 s of white noise + 30 s tone (with shock during the final 2 s) + 30 s white noise + 30 s tone (with shock during the final 2 s) + 30 s white noise. Each rat underwent the aversive conditioning procedure only once.

The STRESSED (*n* = 6) and STRESSED + CE-123 (*n* = 7) groups received paired CS–US presentations during the conditioning session (PND30), whereas the NON-STRESSED (*n* = 6) and NON-STRESSED + CE-123 (*n* = 6) groups were exposed to the auditory CS alone without foot shock. Following conditioning, all animals remained undisturbed for the next 11 days (PND31–41).

#### 4.3.3. Fear Reminder Session (Context B) (PND42)

On PND42, a fear reminder session was conducted in context B, which differed from the original conditioning context (context A). The chamber was modified by introducing checkered-pattern walls, a metal-grid floor with wider spacing between the bars, and the absence of the auditory cue. To further distinguish context B from context A, the chamber was cleaned with a different disinfectant (Surfanios, Laboratoires Anios, Lezennes, France) after each animal, providing a distinct olfactory cue.

The reminder session was designed to reactivate the previously acquired conditioned fear memory without reproducing the original conditioning protocol. During this session, all animals received three low-intensity foot shocks (0.4 mA, 1 s), delivered without presentation of the conditioned auditory stimulus. The protocol consisted of 30 s of white noise followed by a 1 s foot shock (0.4 mA), two consecutive 70 s intervals each ending with a 1 s foot shock (0.4 mA), and a final 30 s period of white noise, resulting in a total session duration of 200 s.

#### 4.3.4. Fear Memory Recall Test (Contexts A and B) (PND43)

In the morning, rats were placed in the conditioning chamber (context A) and exposed to the conditioned auditory stimulus (30 s tone) without foot shock. The session lasted 150 s (2.5 min) and was used to assess recall of the original CS–US association acquired during fear conditioning.

Several hours later, the animals were placed in context B for a 150 s session without foot shock. This session assessed the animals’ behavioral response to the reminder context following memory reactivation.

#### 4.3.5. Elevated Plus Maze Test (PND44)

Anxiety-like behavior was assessed using the EPM test, as previously described [21]. The apparatus consisted of a black-painted wooden maze elevated 50 cm above the floor, with four arms arranged in a cross and connected by a central platform. Two opposite arms were open (50 × 10 cm), whereas the remaining two arms were enclosed by opaque walls (50 × 10 × 40 cm). The test was performed in a quiet, dark room under dim, uniform illumination provided by a 15 W light source positioned 80 cm above the maze. Each rat was placed individually on the central platform facing an open arm and allowed to explore the maze freely for 5 min. Behavior was recorded using a video camera and analyzed with the ANY-maze video tracking system (version 6.3, Stoelting Co., Wood Dale, IL, USA). The following parameters were analyzed: (i) time spent in the open arms and (ii) the number of entries into the open and closed arms. The total distance traveled during the test was also measured.

Between animals, the maze was cleaned with. 10% ethanol to eliminate residual olfactory cues.

#### 4.3.6. Fear Extinction Procedure (PND46–49)

Following the recall test, fear extinction training was initiated. Extinction was conducted in the same sound-attenuating chamber (context A) used during the initial conditioning session. Each extinction session lasted 150 s (2.5 min) and consisted of two presentations of the conditioned auditory stimulus (30 s tone), separated by 30 s intervals of white noise, followed by a final 30 s period of white noise.

Rats from both the STRESSED and NON-STRESSED groups received intraperitoneal injections of CE-123 or vehicle once daily for four consecutive days, administered 30 min before each extinction session. During extinction training, freezing behavior, defined as complete immobility except for respiratory movements, was quantified as an index of conditioned fear expression. A progressive reduction in freezing across extinction sessions was interpreted as successful extinction learning. For descriptive purposes, successful extinction was operationally defined at the group level as a reduction in mean freezing to ≤30% of the mean freezing level recorded during the first extinction session. This criterion was not applied to individual animals and was not used to determine their inclusion in subsequent analyses. All animals completed the full extinction protocol and were retained in the subsequent behavioral and proteomic analyses regardless of their individual extinction performance.

#### 4.3.7. Fear Reinstatement Session (PND50)

Twenty-four hours after the completion of extinction training, animals underwent a fear reinstatement/challenge session in context A. The purpose of this session was to assess the robustness of the extinction-related reduction in conditioned fear following re-exposure to a low-intensity aversive stimulus.

Rats were re-exposed to context A and presented with the conditioned auditory stimulus together with a low-intensity foot shock (0.15 mA). This stimulus intensity was selected because it does not evoke a measurable behavioral response in naïve animals that have not previously undergone fear conditioning.

The session lasted 150 s (2.5 min) and consisted of the following sequence: 30 s of white noise, 30 s tone (with a 0.15 mA foot shock delivered during the final 2 s), 30 s of white noise, 30 s tone (with a 0.15 mA foot shock delivered during the final 2 s), and a final 30 s period of white noise. This procedure was used to determine whether the reduced fear response established during extinction persisted under conditions capable of reinstating conditioned fear.

#### 4.3.8. Final Behavioral Assessment and Tissue Collection (PND51)

**Figure 7:**
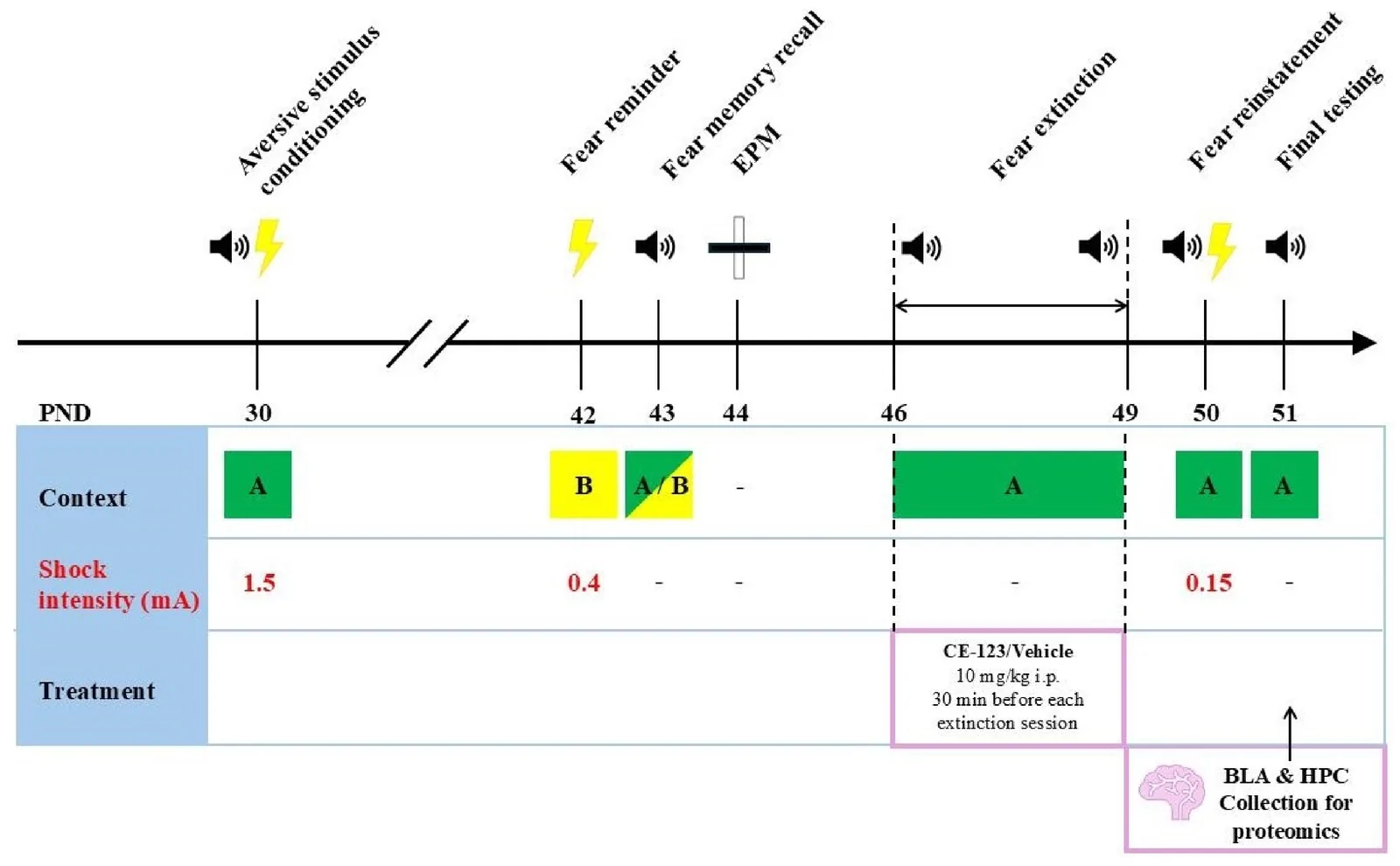
Experimental design illustrating the sequence of behavioral procedures, CE-123 treatment, and tissue collection for proteomic analyses. PND, postnatal day; EPM, elevated plus maze; BLA, basolateral amygdala; HPC, hippocampus.

The final behavioral assessment was performed in context A without delivery of a foot shock. This shock-free test was used to assess conditioned fear expression following the PND50 reinstatement/challenge and to determine whether the extinction-related reduction in freezing persisted in the absence of an acute aversive stimulus. Immediately after completion of behavioral testing, animals were euthanized by decapitation, and the brains were rapidly removed and placed on an ice-cooled dissection surface. Bilateral samples of the hippocampal formation and basolateral amygdala were manually microdissected using anatomical landmarks identified according to the rat brain atlas of Paxinos and Watson [42]. The hippocampal samples included the dorsal and intermediate portions of the hippocampal formation, comprising the CA1 and CA3 fields and dentate gyrus, collected over an approximate rostrocaudal range of −2.0 to −6.0 mm relative to Bregma. The BLA samples encompassed the lateral and basal amygdaloid nuclei and were collected over an approximate rostrocaudal range of −1.8 to −3.6 mm relative to Bregma. Because tissue collection was performed by manual microdissection rather than stereotaxic tissue punching, these coordinates indicate the approximate anatomical extent of the collected regions. Samples from the left and right hemispheres were pooled for each animal, immediately snap-frozen, and stored at −80 °C until proteomic analysis. A schematic representation of the approximate tissue collection sites is provided in Figure S6. The experimental timeline of the behavioral procedures is presented in Figure 7.

### 4.4. Proteomic Analysis

All animals were included in behavioral analyses, whereas the same animals were subsequently used for proteomic analyses.

#### 4.4.1. Sample Preparation

For proteomic analyses, tissues were ultrasonically homogenized (UP100H; Hielscher Ultrasonics, Teltow, Germany; 30 s, 80 W, 50% duty cycle) in 1 mL of 50 mM ammonium bicarbonate buffer (AMBIC, pH 8.0) supplemented with 4% (*w*/*v*) sodium dodecyl sulfate (SDS) and cOmplete™ Protease Inhibitor Cocktail (all reagents from Merck-Millipore, Darmstadt, Germany), prepared according to the manufacturer’s instructions. Homogenates were centrifuged at 24,000× *g* for 10 min at room temperature (Eppendorf 5804R, Eppendorf GmbH, Leipzig, Germany), and the supernatants were collected.

Protein concentration was determined using the bicinchoninic acid (BCA) assay with a Spectrostar Nano microplate reader (BMG Labtech, Aylesbury, UK). All samples were adjusted to a final protein concentration of 0.5 mg/mL (50 μg protein in 100 μL).

Protein disulfide bonds were reduced with 20 mM dithiothreitol (DTT) for 30 min at 37 °C, followed by alkylation with 40 mM iodoacetamide (IAA) for 30 min at room temperature in the dark (both reagents from Merck-Millipore, Darmstadt, Germany).

Samples were subsequently processed using the KingFisher™ Flex automated purification system (Thermo Fisher Scientific, Bremen, Germany). Proteins were captured on MagReSyn^®^ Amine magnetic beads (Resyn Biosciences, Edenvale, South Africa), purified to remove SDS and other contaminants, and digested with Trypsin Gold (Promega, Madison, WI, USA; 2.5 μg per sample). Proteolytic digestion was carried out for 4 h at 50 °C.

Following digestion, peptide concentration was determined using the Quantitative Fluorometric Peptide Assay (Thermo Fisher Scientific/Pierce, cat. no. 23290). Samples were supplemented with acetonitrile (ACN) and formic acid to obtain final concentrations of 4% ACN and 0.1% formic acid, while maintaining a peptide concentration of at least 0.2 μg/μL.

Injection volumes were individually adjusted to introduce 0.8 μg of tryptic peptides into the nanoLC–MS/MS system. Each biological sample was analyzed in duplicate LC–MS/MS runs, and the injection order was randomized to minimize potential batch effects and instrumental drift.

#### 4.4.2. nanoLC–MS/MS

Peptide separation and LC–MS/MS analysis were performed as previously described [43] using an Ultimate™ 3000 nanoLC system coupled to an Orbitrap Exploris™ 240 mass spectrometer equipped with a Nanospray Flex™ ion source (Thermo Fisher Scientific, Bremen, Germany). Instrument control and data acquisition were performed using Xcalibur software (version 4.5.445.18).

Peptides were first loaded onto an Acclaim™ PepMap™ Neo trap column (5 mm × 0.5 mm ID) and subsequently separated on an Acclaim™ PepMap™ C18 analytical column (50 cm × 75 μm ID, reversed-phase C18) (both columns from Thermo Fischer Scientific, Lithuania). The mobile phases consisted of (A) water containing 0.1% formic acid (*v*/*v*) and (B) acetonitrile containing 0.1% formic acid (*v*/*v*). The LC gradient was as follows: 0–5 min, 12% B; 5–75 min, linear increase to 34% B; 75–78 min, increase to 70% B; 78–83 min, hold at 70% B; 83–85 min, decrease to 12% B; and 85–90 min, equilibration at 12% B.

Full-scan MS spectra were acquired over an m/z range of 350–1150 at a resolution of 60,000. DIA MS/MS spectra were acquired at a resolution of 15,000 using variable-width isolation windows. The Automatic Gain Control (AGC) target was set to 1 × 10^6^ for MS1 scans and 200% for MS2 scans, with a maximum injection time of 100 ms. Higher-energy collisional dissociation (HCD) was performed using a normalized collision energy (NCE) of 27. A total of 40 variable-width DIA isolation windows with a 1 Da overlap was applied across the analyzed m/z range, resulting in a total cycle time of approximately 2.5 s. MS data acquisition was performed between 6.0 and 89.0 min of the 90 min LC gradient.

### 4.5. Statistical Analysis

#### 4.5.1. Behavioral Data Analysis

Behavioral data were analyzed using GraphPad Prism (version 8.3.0; GraphPad Software, San Diego, CA, USA). Statistical comparisons were performed using an unpaired Student’s *t*-test or two-way analysis of variance (two-way ANOVA), followed by Bonferroni’s post hoc test where appropriate. Differences were considered statistically significant at *p* < 0.05. Data are presented as the mean ± SEM.

#### 4.5.2. Proteomic Data Analysis

Raw mass spectrometry (*.raw) files were processed using DIA-NN (version 2.1.0, Academic version). A library-free workflow was applied using the UniProt reference proteome UP000002494 (Rattus norvegicus) without prior DDA or gas-phase fractionation. DIA-NN settings included N-terminal methionine excision, carbamidomethylation of cysteine as a fixed modification, trypsin/P specificity, peptide lengths of 6–40 amino acids, precursor charge states of 2–5, precursor m/z range of 350–1151, and fragment ion m/z range of 200–2000. Precursor-level false discovery rate (FDR) was controlled at 1%, and protein quantification was performed using the High Accuracy strategy.

Downstream statistical analysis was performed using Perseus (version 2.0.8.1) with an identical processing pipeline applied to all comparisons. Protein intensity values were log_2_-transformed, median-normalized, and filtered to retain proteins with at least 70% valid values across samples. Differential protein abundance was determined using a two-sample Student’s *t*-test. Statistical significance was assessed using the Benjamini–Hochberg false discovery rate (FDR), with an adjusted *p* value < 0.05 considered significant. Proteins meeting this criterion are listed in Supplementary Tables S1 and S2.

For visualization purposes, statistically processed datasets were exported to VolcanoseR [44]. Because of the relatively small sample size (*n* = 6 per group), volcano plots were generated using a relaxed threshold of *p* = 0.02 (−log_10_(*p*) = 1.7) together with an absolute log_2_(fold change) > 0.5 to facilitate visualization of broader proteomic trends. These thresholds were used exclusively for graphical presentation, whereas statistical inference was based on FDR-adjusted analyses. Further details of the Perseus workflow have been described previously [45].

The mass spectrometry proteomics data have been deposited in the ProteomeXchange Consortium via the PRIDE partner repository [46] under the dataset identifier PXD075812.

## 5. Conclusions

The present study demonstrates that repeated administration of the selective DAT inhibitor CE-123 during extinction training reduced persistent fear responses in stressed adolescent male rats. Proteomic alterations were substantially more pronounced in the BLA than in the HPC, identifying the BLA as a major site of molecular remodeling associated with persistent fear and its pharmacological modulation. CE-123 did not simply restore the non-stressed proteomic profile but produced a distinct pattern of changes involving pathways related to synaptic organization, neuronal excitability, RNA processing, mitochondrial function, and cellular metabolism.

Because the proteomic findings are associative, were obtained at a single time point, and were not independently validated, their causal contribution to the behavioral effects of CE-123 remains to be established. Nevertheless, the convergence of the behavioral and molecular results supports further investigation of selective DAT inhibition as a potential approach to facilitating fear extinction. Studies incorporating both sexes, additional components of the fear-regulatory circuit, temporally resolved proteomic analyses, and functional validation of selected proteins will be necessary to define the mechanisms and potential therapeutic relevance of CE-123 in stress-related disorders.

## Figures and Tables

**Table 1:** The five most significantly upregulated and downregulated proteins identified in each experimental group comparison in the hippocampus (HPC) and basolateral amygdala (BLA). Fold changes for proteins marked in bold excessed a 2-fold change relative to the respective control group. Detailed results for all differentially abundant proteins are provided in Supplementary Tables S1 and S2.

Comparison:	Hippocampus			Basolateral Amygdala		
	Type of Regulation	Top 5 Proteins		Type of Regulation	Top 5 Proteins	
		Up-Reg.	Down-Reg.		Up-Reg.	Down-Reg.
**STRESSED** **vs.** **NON-STRESSED**	up-regulation: - neuronal plasticity - cellular metabolism	D3ZEH2_RAT Q5XI38_RAT **KPCD_RAT** NIT2_RAT A0A0G2JVT8_RAT	CPLX2_RAT CLYBL_RAT SEM6B_RAT PTBP3_RAT DOCK9_RAT	up-regulation: - axonal remodelling - glutamatergic synapse activity - postsynaptic changes	HPT_RAT D4AA77_RAT D3ZUU5_RAT D3ZH14_RAT **NRP2_RAT**	**NU2M_RAT** **P2RY1_RAT** **TBB2B_RAT** CLYBL_RAT D3ZGL1_RAT
**STRESSED** **+ CE123** **vs.** **STRESSED**	down-regulation: - protein folding - mitochondrial activation	**KCRM_RAT** CK5P1_RAT **M0RCZ9_RAT** CPLX2_RAT MBLC1_RAT	A0A0G2K5S7_RAT CYC_RAT FXYD6_RAT **AKCL2_RAT** D4A7Z5_RAT	down-regulation - ribosomal activity - mitochondrial activation - GTP hydrolysis - increased RNA metabolism	**M0RCZ9_RAT** **KCRM_RAT** GTPB3_RAT JPH4_RAT F1LNJ1_RAT	**RL32_RAT** **RL15_RAT** SGTA_RAT **D3ZUP5_RAT** CC127_RAT
**NON-STRESSED** **+ CE123** **vs.** **NON-STRESSED**	down-regulation - mitochondrial deactivation - cytoskeleton remodelling	A0A8I6AHL9_RAT FMC1_RAT **KCRM_RAT** S41A3_RAT GNTK_RAT	CYC_RAT VLDLR_RAT **AKCL2_RAT** MARK1_RAT MBNL1_RAT	down-regulation - mitochondrial ATP synthesis - electron transport chain	**M0RCZ9_RAT** **KCRM_RAT** **PRVA_RAT** SNP23_RAT HOME2_RAT	**RL32_RAT** RL35_RAT RL6_RAT SGTA_RAT RL18_RAT

**Table 2:** Study group design, including fear model induction (Yes/No) and assigned treatment (Vehicle or CE-123).

Fear Conditioning Model	Treatment	Group Designation
No	Vehicle	NON-STRESSED
	CE-123	NON-STRESSED + CE-123
Yes	Vehicle	STRESSED
	CE-123	STRESSED + CE-123

## Data Availability

Proteomic data generated in this study are deposited in the PRIDE database, administered by EMBL-EBI, under accession number PXD075812. The behavioral data generated in this study are available from the corresponding author upon reasonable request.

## Supplementary Materials

The following supporting information can be downloaded at: https://www.mdpi.com/article/10.3390/ijms27188352/s1.

## Abbreviations

The following abbreviations are used in this manuscript:

ACN	Acetonitrile
AGC	Automatic Gain Control
AMBIC	Ammonium bicarbonate
BCA	Bicinchoninic acid
BLA	Basolateral amygdala
CE-123	5-((benzhydrylsulfinyl)methyl)thiazole
CS	Conditioned stimulus
DAT	Dopamine transporter
DIA	Data-independent acquisition
DMSO	Dimethyl sulfoxide
DTT	Dithiothreitol
EPM	Elevated plus maze
FDR	False discovery rate
GO	Gene Ontology
HCD	Higher-energy collisional dissociation
HPC	Hippocampus
HPLC	High-performance liquid chromatography
IAA	Iodoacetamide
LC–MS/MS	Liquid chromatography–tandem mass spectrometry
NIH	National Institutes of Health
PCA	Principal component analysis
PFC	Prefrontal cortex
PND	Postnatal day
PTSD	Post-traumatic stress disorder
SDS	Sodium dodecyl sulfate
SEM	Standard error of the mean
SEFL	Stress-enhanced fear learning
SSRIs	Selective serotonin reuptake inhibitors
US	Unconditioned stimulus
