# Supplementary material for: Selective Dopamine Transporter Inhibition with CE-123 Promotes Fear Extinction and Alters the Basolateral Amygdala Proteome Following Fear Extinction in Adolescent Male Rats
Source: Int J Mol Sci. 2026 Sep 19;27(18):8352. doi: 10.3390/ijms27188352 (PMC13607571; doi:10.3390/ijms27188352)
Supplement: Supplementary file 1 [file ijms-27-08352-s001.zip › Figure_S1.pdf]

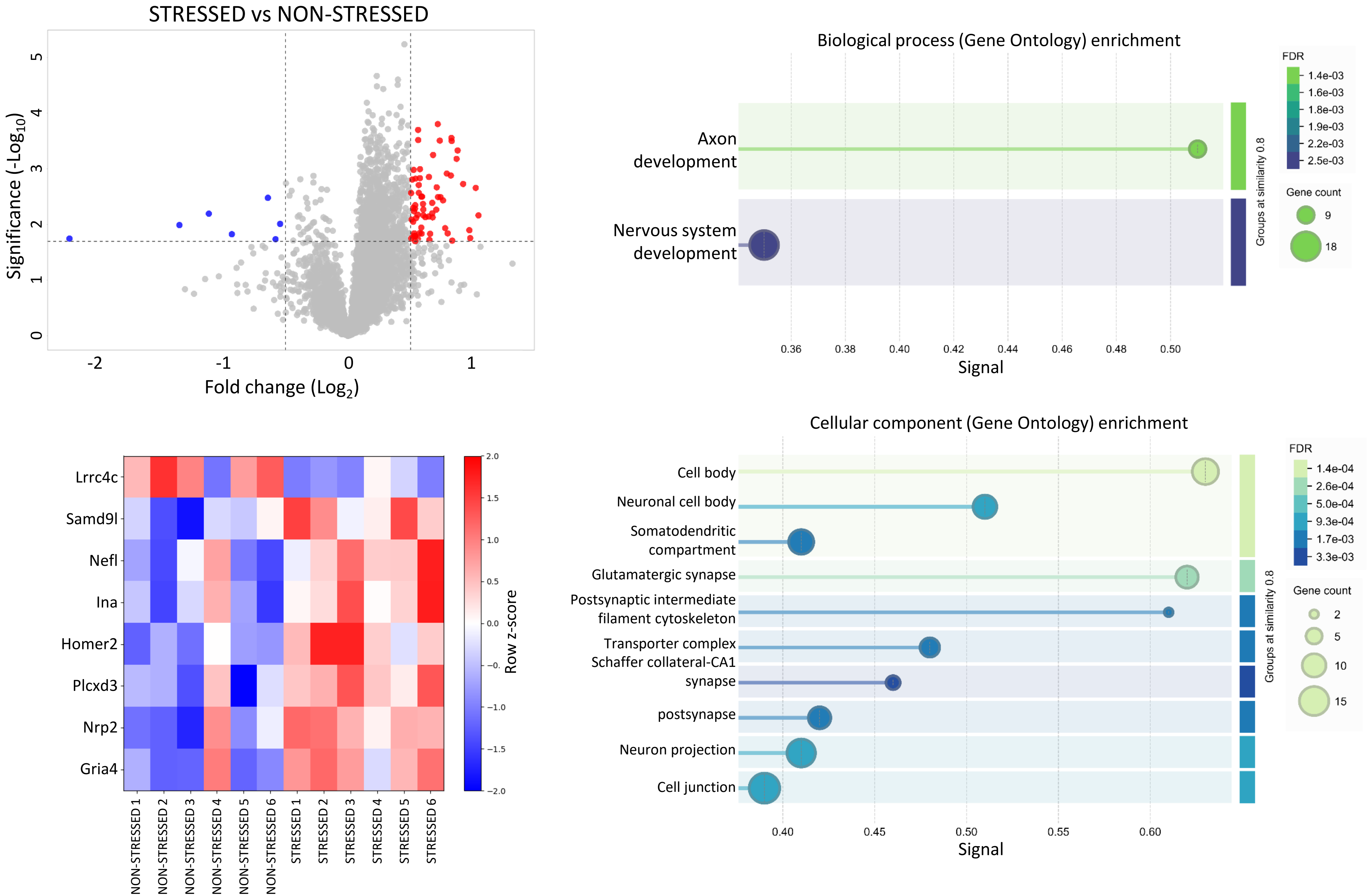

STRESSED vs NON-STRESSED
Significance (-Log10)
Fold change (Log2)
Biological process (Gene Ontology) enrichment
Axon development
Nervous system development
Signal
FDR
1.4e-03
1.6e-03
1.8e-03
1.9e-03
2.2e-03
2.5e-03
Gene count
9
18
Groups at similarity 0.8
Lrrc4c
Samd9l
Nefl
Ina
Homer2
Plcxd3
Nrp2
Gria4
NON-STRESSED 1
NON-STRESSED 2
NON-STRESSED 3
NON-STRESSED 4
NON-STRESSED 5
NON-STRESSED 6
STRESSED 1
STRESSED 2
STRESSED 3
STRESSED 4
STRESSED 5
STRESSED 6
Row z-score
Cellular component (Gene Ontology) enrichment
Cell body
Neuronal cell body
Somatodendritic compartment
Glutamatergic synapse
Postsynaptic intermediate filament cytoskeleton
Transporter complex
Schaffer collateral-CA1 synapse
postsynapse
Neuron projection
Cell junction
Signal
FDR
1.4e-04
2.6e-04
5.0e-04
9.3e-04
1.7e-03
3.3e-03
Gene count
2
5
10
15
Groups at similarity 0.8
