# Supplementary material for: Selective Dopamine Transporter Inhibition with CE-123 Promotes Fear Extinction and Alters the Basolateral Amygdala Proteome Following Fear Extinction in Adolescent Male Rats
Source: Int J Mol Sci. 2026 Sep 19;27(18):8352. doi: 10.3390/ijms27188352 (PMC13607571; doi:10.3390/ijms27188352)
Supplement: Supplementary file 1 [file ijms-27-08352-s001.zip › Figure_S2.pdf]

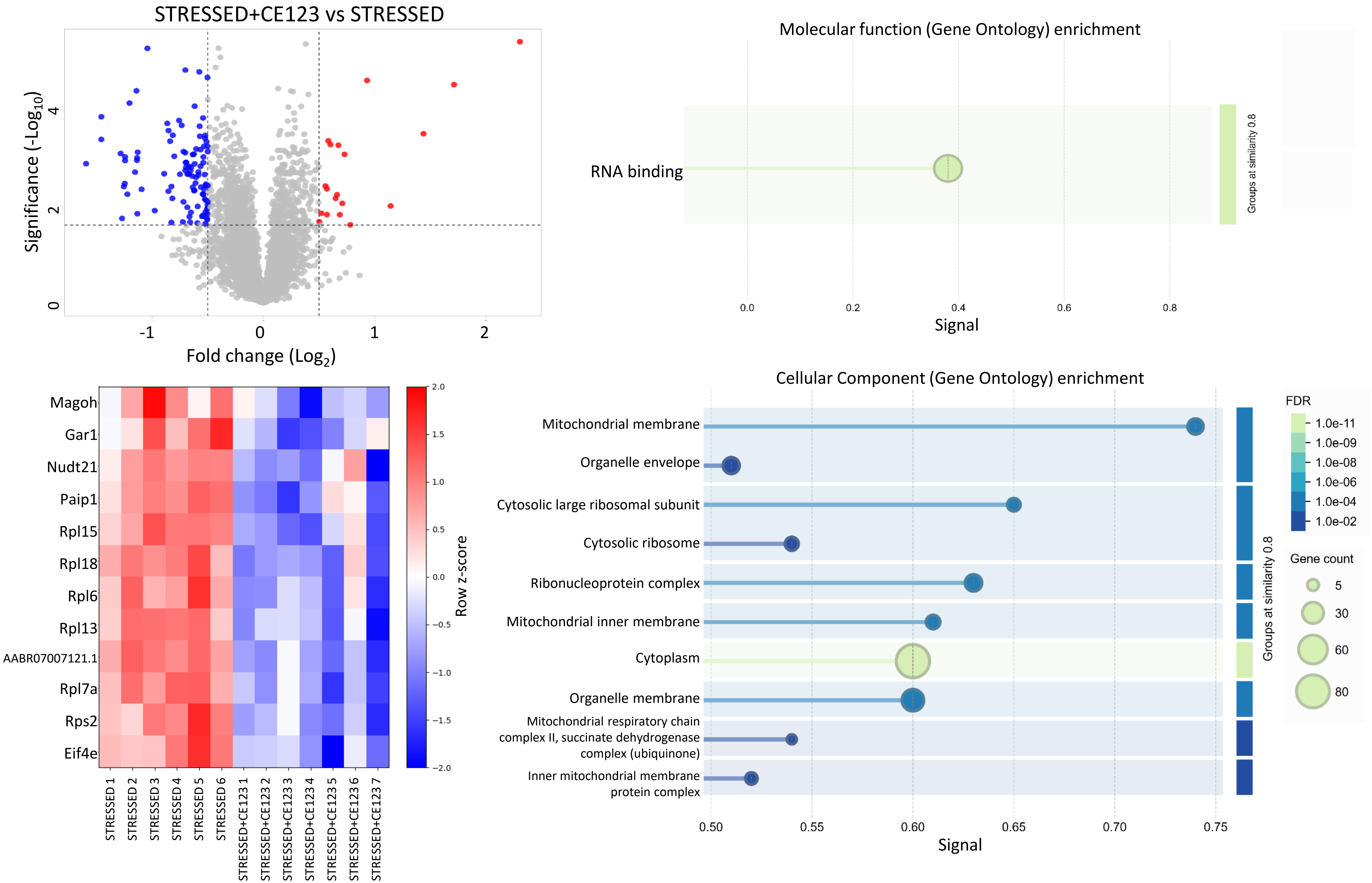
STRESSED+CE123 vs STRESSED
Significance (-Log10)
Fold change (Log2)
0
2
4
-1
0
1
2
Molecular function (Gene Ontology) enrichment
RNA binding
Groups at similarity 0.8
0.0
0.2
0.4
0.6
0.8
Signal
Magoh
Gar1
Nudt21
Paip1
Rpl15
Rpl18
Rpl6
Rpl13
AABR07007121.1
Rpl7a
Rps2
Eif4e
STRESSED 1
STRESSED 2
STRESSED 3
STRESSED 4
STRESSED 5
STRESSED 6
STRESSED+CE123 1
STRESSED+CE123 2
STRESSED+CE123 3
STRESSED+CE123 4
STRESSED+CE123 5
STRESSED+CE123 6
STRESSED+CE123 7
Row z-score
2.0
1.5
1.0
0.5
0.0
−0.5
−1.0
−1.5
−2.0
Cellular Component (Gene Ontology) enrichment
Mitochondrial membrane
Organelle envelope
Cytosolic large ribosomal subunit
Cytosolic ribosome
Ribonucleoprotein complex
Mitochondrial inner membrane
Cytoplasm
Organelle membrane
Mitochondrial respiratory chain complex II, succinate dehydrogenase complex (ubiquinone)
Inner mitochondrial membrane protein complex
0.50
0.55
0.60
0.65
0.70
0.75
Signal
Groups at similarity 0.8
FDR
1.0e-11
1.0e-09
1.0e-08
1.0e-06
1.0e-04
1.0e-02
Gene count
5
30
60
80
