# Supplementary material for: Selective Dopamine Transporter Inhibition with CE-123 Promotes Fear Extinction and Alters the Basolateral Amygdala Proteome Following Fear Extinction in Adolescent Male Rats
Source: Int J Mol Sci. 2026 Sep 19;27(18):8352. doi: 10.3390/ijms27188352 (PMC13607571; doi:10.3390/ijms27188352)
Supplement: Supplementary file 1 [file ijms-27-08352-s001.zip › Figure_S3.pdf]

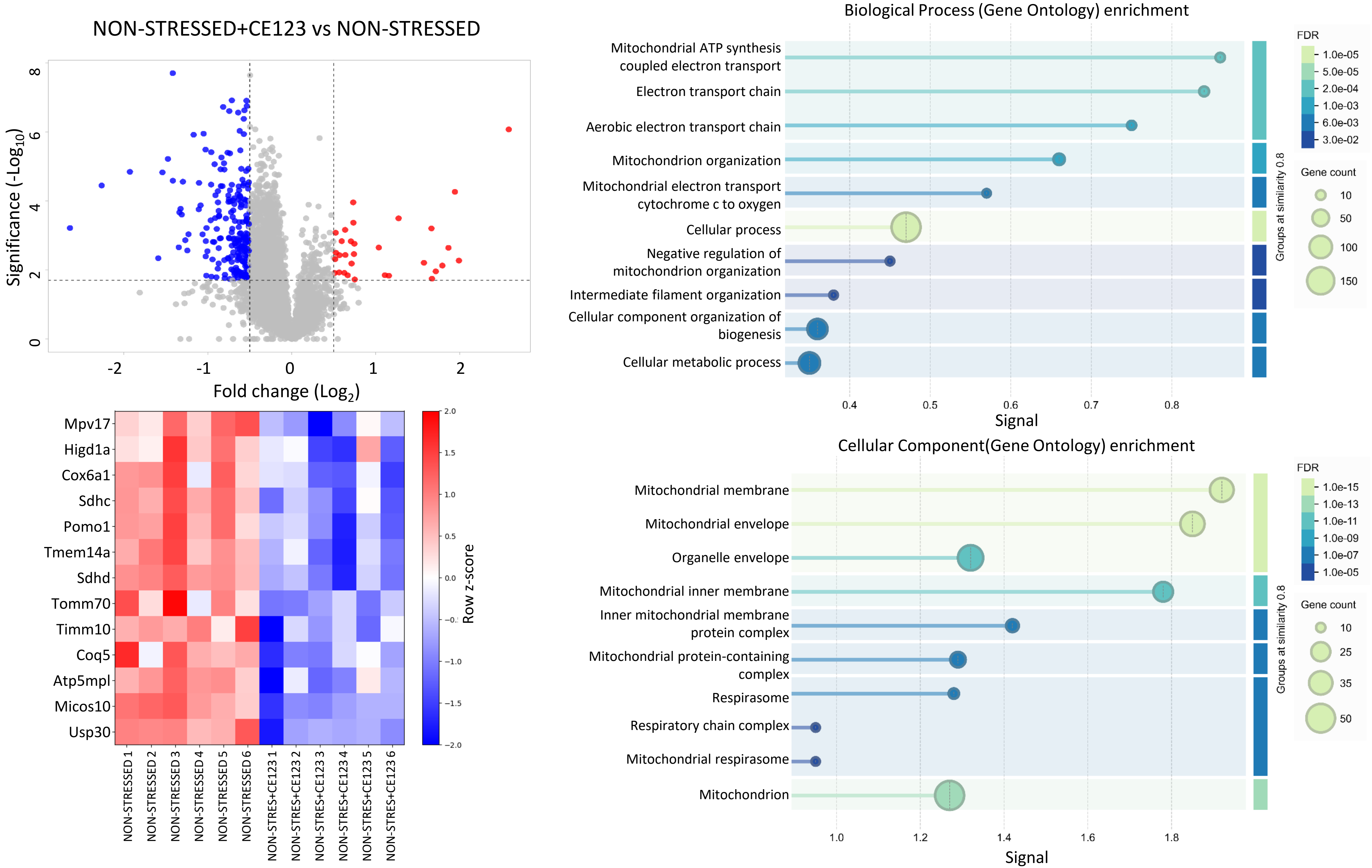

NON-STRESSED+CE123 vs NON-STRESSED
Significance (-Log10)
Fold change (Log2)
0
2
4
6
8
-2
-1
0
1
2
Mpv17
Higd1a
Cox6a1
Sdhc
Pomo1
Tmem14a
Sdhd
Tomm70
Timm10
Coq5
Atp5mpl
Micos10
Usp30
NON-STRESSED 1
NON-STRESSED 2
NON-STRESSED 3
NON-STRESSED 4
NON-STRESSED 5
NON-STRESSED 6
NON-STRES+CE123 1
NON-STRES+CE123 2
NON-STRES+CE123 3
NON-STRES+CE123 4
NON-STRES+CE123 5
NON-STRES+CE123 6
Row z-score
2.0
1.5
1.0
0.5
0.0
−1.0
−1.5
−2.0
Biological Process (Gene Ontology) enrichment
Mitochondrial ATP synthesis coupled electron transport
Electron transport chain
Aerobic electron transport chain
Mitochondrion organization
Mitochondrial electron transport cytochrome c to oxygen
Cellular process
Negative regulation of mitochondrion organization
Intermediate filament organization
Cellular component organization of biogenesis
Cellular metabolic process
0.4
0.5
0.6
0.7
0.8
Signal
Groups at similarity 0.8
FDR
1.0e-05
5.0e-05
2.0e-04
1.0e-03
6.0e-03
3.0e-02
Gene count
10
50
100
150
Cellular Component(Gene Ontology) enrichment
Mitochondrial membrane
Mitochondrial envelope
Organelle envelope
Mitochondrial inner membrane
Inner mitochondrial membrane protein complex
Mitochondrial protein-containing complex
Respirasome
Respiratory chain complex
Mitochondrial respirasome
Mitochondrion
1.0
1.2
1.4
1.6
1.8
Signal
Groups at similarity 0.8
FDR
1.0e-15
1.0e-13
1.0e-11
1.0e-09
1.0e-07
1.0e-05
Gene count
10
25
35
50
