# Supplementary material for: Selective Dopamine Transporter Inhibition with CE-123 Promotes Fear Extinction and Alters the Basolateral Amygdala Proteome Following Fear Extinction in Adolescent Male Rats
Source: Int J Mol Sci. 2026 Sep 19;27(18):8352. doi: 10.3390/ijms27188352 (PMC13607571; doi:10.3390/ijms27188352)
Supplement: Supplementary file 1 [file ijms-27-08352-s001.zip › Figure_S4.pdf]

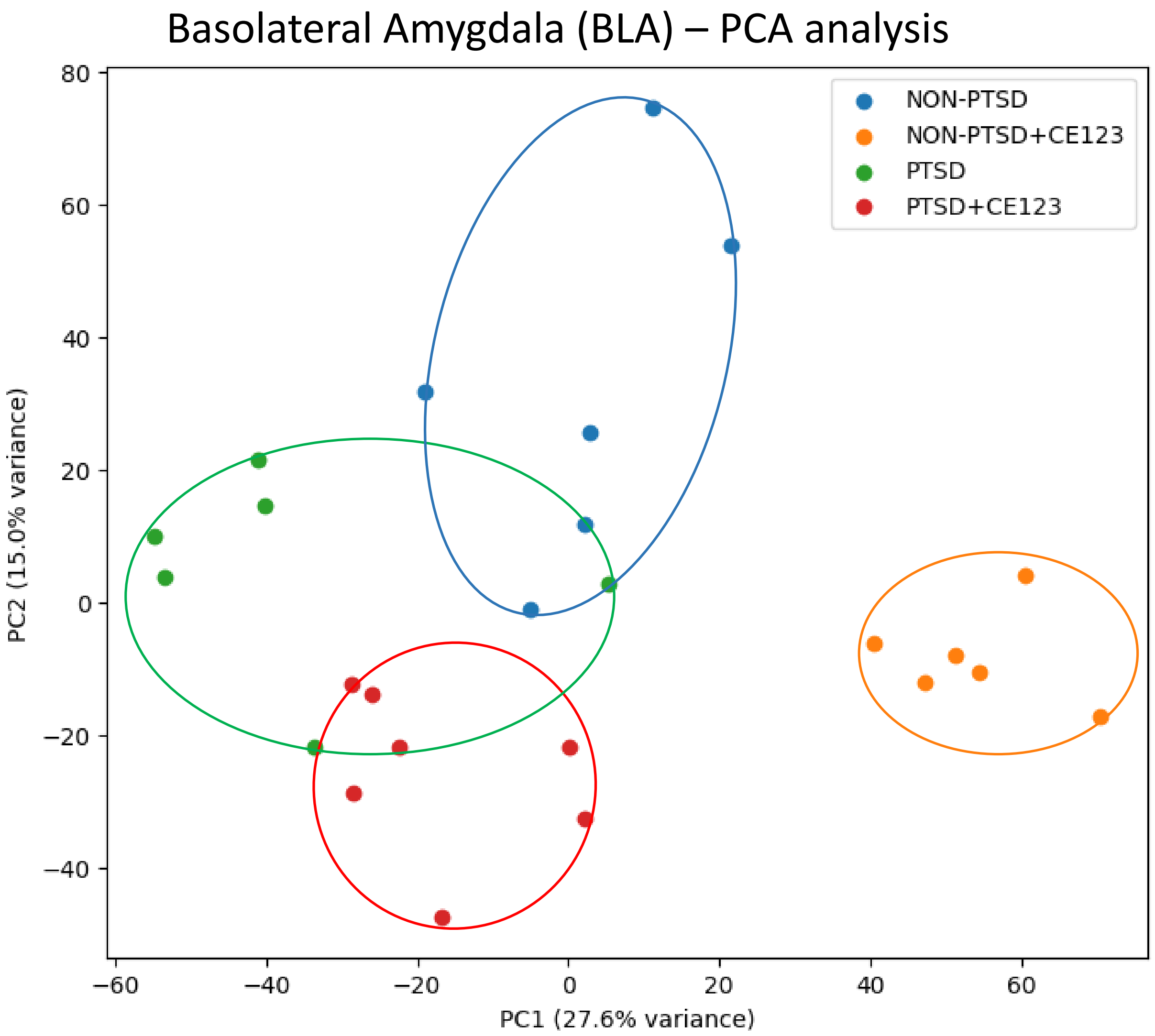
Basolateral Amygdala (BLA) – PCA analysis
NON-PTSD
NON-PTSD+CE123
PTSD
PTSD+CE123
PC2 (15.0% variance)
PC1 (27.6% variance)
80
60
40
20
0
−20
−40
−60
−40
−20
0
20
40
60

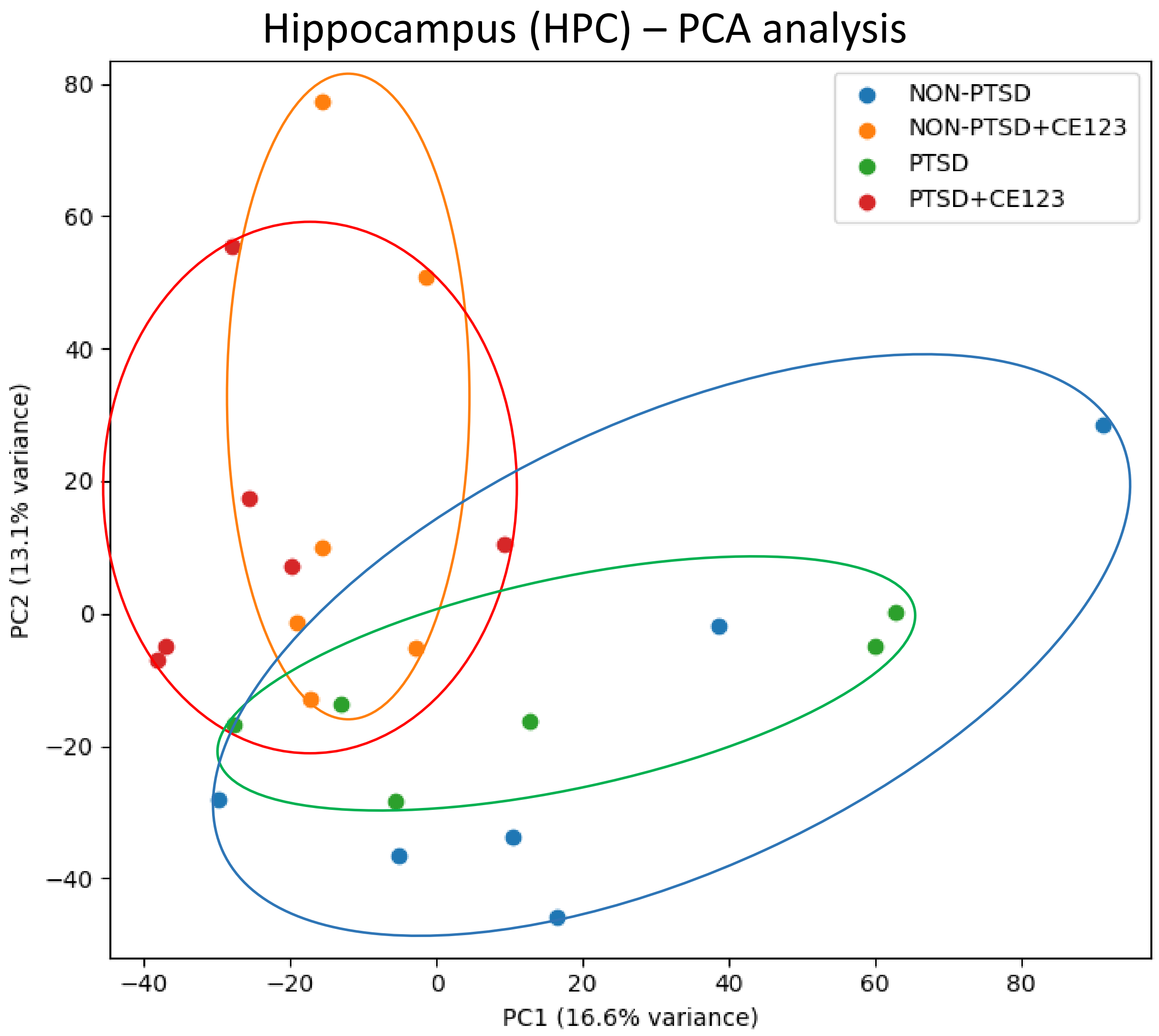
Hippocampus (HPC) – PCA analysis
NON-PTSD
NON-PTSD+CE123
PTSD
PTSD+CE123
PC2 (13.1% variance)
PC1 (16.6% variance)
80
60
40
20
0
−20
−40
−40
−20
0
20
40
60
80
