# Supplementary material for: Selective Dopamine Transporter Inhibition with CE-123 Promotes Fear Extinction and Alters the Basolateral Amygdala Proteome Following Fear Extinction in Adolescent Male Rats
Source: Int J Mol Sci. 2026 Sep 19;27(18):8352. doi: 10.3390/ijms27188352 (PMC13607571; doi:10.3390/ijms27188352)
Supplement: Supplementary file 1 [file ijms-27-08352-s001.zip › Figure_S5.pdf]

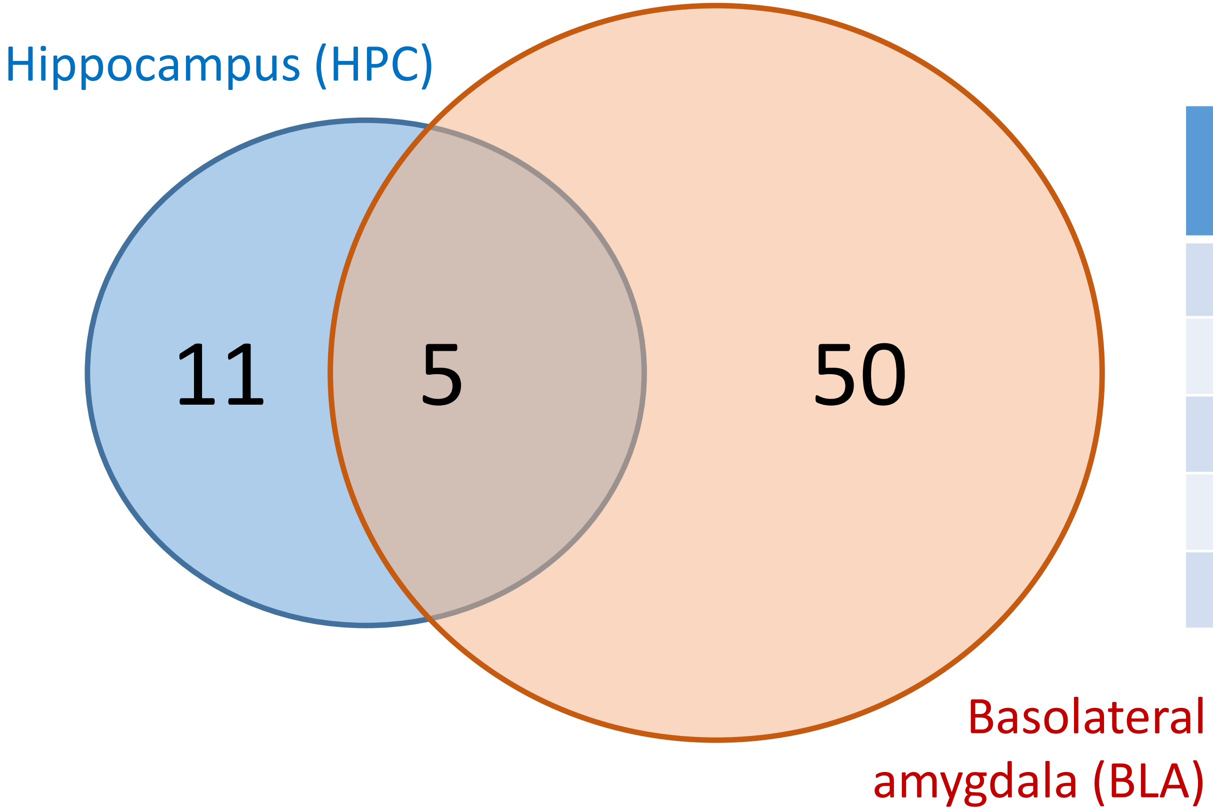


| overlapping protein | HPC/fold change | BLA/fold change |
|---|---|---|
| NIT2_RAT | ↑ / 0.932 | ↑ / 0.921 |
| CAH1_RAT | ↑ / 0.580 | ↑ / 0.599 |
| A0A0G2JVT8_RAT | ↑ / 0.696 | ↑ / 0.673 |
| HPT_RAT | ↑ / 0.666 | ↑ / 0.829 |
| CLYBL_RAT | ↓ / -0.988 | ↓ / -0.929 |
