# Supplementary material for: Selective Dopamine Transporter Inhibition with CE-123 Promotes Fear Extinction and Alters the Basolateral Amygdala Proteome Following Fear Extinction in Adolescent Male Rats
Source: Int J Mol Sci. 2026 Sep 19;27(18):8352. doi: 10.3390/ijms27188352 (PMC13607571; doi:10.3390/ijms27188352)
Supplement: Supplementary file 1 [file ijms-27-08352-s001.zip › Figure_S6.pdf]

Schematic representation of tissue collection sites

A. Hippocampus (HPC)
Representative coronal section: approximately Bregma −3.5 mm

B. Basolateral amygdala (BLA)
Representative coronal section: approximately Bregma −2.5 mm

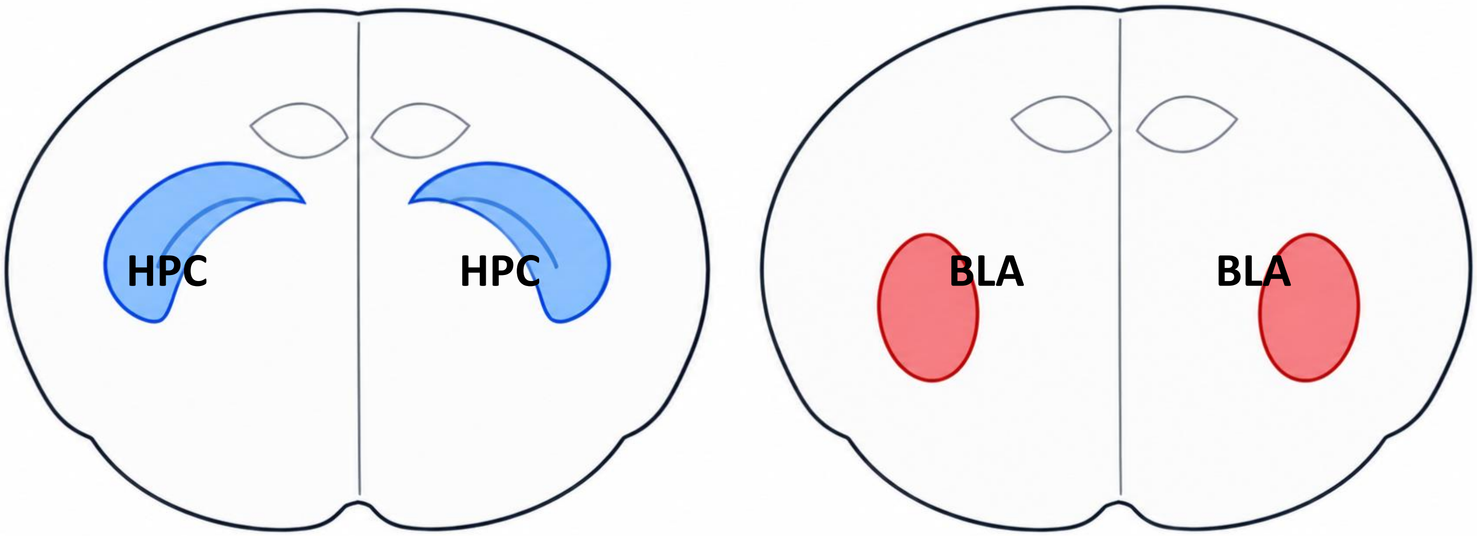

Bilateral manual microdissection guided by anatomical landmarks in the Paxinos and Watson rat brain atlas.
